# Mechanistic models with experimental comparison for microwave-assisted lyophilization in a vial

**DOI:** 10.1186/s41120-026-00173-3

**Published:** 2026-07-06

**Authors:** Isaac S. Wheeler, Ahmad Darwish, Vivek Narsimhan, Alina A. Alexeenko

**Affiliations:** 1 Davidson School of Chemical Engineering, Purdue University, 480 Stadium Mall Drive, West Lafayette, 47907 Indiana USA; 2 School of Aeronautics and Astronautics, Purdue University, 701 W Stadium Ave, West Lafayette, 47907 Indiana USA; 3 Birck Nanotechnology Center, Purdue University, 1205 Mitch Daniels Boulevard, West Lafayette, 47907 Indiana USA

**Keywords:** Heat and mass transfer, Lyophilization, Microwave heating, Mechanistic model

## Abstract

Microwave heating has been shown to accelerate pharmaceutical lyophilization but robust process models, crucial to ensuring that the process is efficient without damaging products, are still scarce. Building on both high-frequency microwave lyophilization experiments and recent modeling efforts, we develop a 2D level set simulation which refines the assumptions made in lumped capacitance (LC) models. The 2D level set model is also capable of integrating information about measured porous structures and accounting for experimental observations where thermocouples are placed imperfectly. Of the 4 empirical parameters in the LC model, we remove one based on level set results and estimate two more by electromagnetic simulation, recommending a new variant of the 0D lumped capacitance model. This LC model is compared to experiments in both microwave-assisted lyophilization and microwave vacuum drying of products in vials, emphasizing that microwave heating of the vial wall and heat conduction from the vial wall to frozen product cannot be neglected. The implementations of the new models, alongside the analysis, are published as open source Julia code.

**Graphical Abstract:**

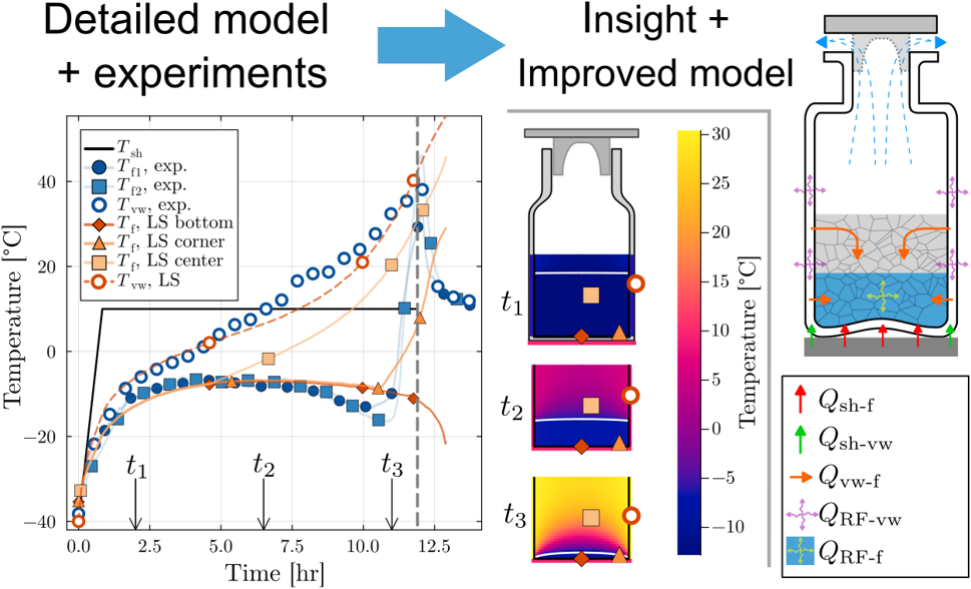

## Introduction

Humility and curiosity are fundamental prerequisites to learning, and even a short chat with Dr. Steve Nail is enough to learn he is an excellent example of both traits. Maintaining that attitude even after a half-century of his research leadership in lyophilization is remarkable—little wonder then that he has continued to learn, contributed so much to the advancement of the field, and cultivated so many fruitful collaborations. We are happy to pay him homage as part of the Steven L. Nail dedicated collection on freeze drying technology.

One of his early contributions (Nail 1980) was to establish that, in pharmaceutical lyophilization, there is a tradeoff between accelerating mass transfer and heat transfer: low chamber pressures increase the driving force for flow of water vapor, but also decrease the heat exchange between the curved bottom of vials and the shelf they rest on. Michael Pikal took this into account in the model for lyophilization (Pikal et al. 1983) that is still widely used today (Tchessalov et al. 2022).

Lyophilization is an increasingly common step in the manufacture of pharmaceuticals (Lyohub 2025), yielding a shelf-stable solid form for many thermally sensitive materials by sublimating away water after freezing. And yet the bulk of lyophilization literature simply deals with the tradeoff between mass and heat transfer, such as by incorporating it into design spaces (Srinivasan et al. 2023). Perhaps surprisingly, although microwave heating is being actively explored as a technique for improving the energy and time efficiency of lyophilizing drugs, not much attention has been drawn to the way that microwave heating can avoid this tradeoff. Microwave (a.k.a. radio-frequency, or RF) electromagnetic heating works by inducing heating throughout the volume of a material without requiring thermal contact by conduction or convection. In the context of pharmaceutical lyophilization, this means directly heating frozen material without a reliance on heat conduction from the underlying shelf. Removing thermal contact as a design constraint for drying processes will likely lead to much more efficient drying processes in future, but the incremental improvement of retrofitting a traditional lyophilization process with microwave heating still serves an important stepping stone as the technology matures.

Recent experimental studies which do so include Gitter et al. (2018, 2019); Hardter et al. (2023a, 2023b), with a general trend that lyophilization proceeds faster when microwave heating is added. Bhambhani et al. (2021) proposed a heuristic model with which they argue that acceleration is due to the heat transfer behavior, i.e. that microwave energy simply combines with the shelf heating to drive sublimation. Alexeenko et al. (2025) presented experimental measurements for high-frequency microwave lyophilization (above 8 GHz), as well as a lumped capacitance (LC) model.

A mechanistic model capable of predicting product temperatures during drying is crucial in developing and applying the technology, since the purpose of lyophilization is to stabilize thermally-sensitive compounds; without a model, the only way to decide how much microwave heating is appropriate is by trial-and-error, an undesirable prospect for high-value drug material. Witkiewicz and Nastaj (2010) presented a 1D model for general microwave-assisted freeze drying, which they compared to experiments on the drying of active carbon and raw beef. They noted that in assuming 1D behavior, “the smaller the diameter of the container is, the more irregularities of the plane moving boundary are observed at the cylindrical wall”. Building on that work, Park et al. (2021) proposed a model (also used in Srisuma et al. (2023)) and compared that model to one experiment from Gitter et al. (2019). Abdelraheem et al. (2022) estimated drying speedup for microwave-assisted lyophilization by adding an empirical microwave heating contribution to the conventional lyophilization model of LyoPRONTO (Shivkumar et al. 2019), but they reported neither measured nor model-predicted temperatures. Alexeenko et al. (2025) introduced a lumped capacitance (LC) model with the capability of describing the temperatures observed in microwave-assisted lyophilization, as well as predicting drying time.

Unlike Witkiewicz and Nastaj (2010), Park et al. (2021) assumed that the temperature of the product during drying is fixed by thermodynamic equilibrium between sublimating ice and the chamber pressure; this assumption has been used elsewhere for conventional lyophilization (e.g. Dryer and Sunderland (1968); Hottot et al. (2006); Jafar and Farid (2003)), on the grounds that approximately constant product temperature suggests a thermodynamic equilibrium between sublimation front and the chamber pressure. While this assumption is appropriate for pure ice and often applied for gravimetric measurements of the heat transfer coefficient, such as in Hottot et al. (2005), this argument neglects the possibility that mass transfer resistance itself plateaus and yields nearly-constant temperatures, as showcased by Milton et al. (1997) for a 5% lactose solution. Foundational theory work (Pikal et al. 1983; Pikal 1985; Velardi and Barresi 2008), current best practice recommendations (Tchessalov et al. 2023), and industrial lyophilization data (Srinivasan et al. 2023) all indicate that for pharmaceutical contexts the dry product’s mass transfer resistance (often denoted $R_p$) should not be neglected without first confirming that its value is small.

The model which sees the most practical use in conventional lyophilization is one-dimensional (Tchessalov et al. 2022; Srinivasan et al. 2023) and assumes that the sublimation front, the boundary between the dry and still-frozen regions, is planar. In Pikal et al. (2005); Velardi and Barresi (2008), 2D models were investigated, and both studies concluded that the predictive capacity of the model is not lost when radial behavior is neglected. However, the experimental results of Alexeenko et al. (2025) showed that, when microwave heating is added, glass temperatures may be much higher than the frozen layer. This induces much more radial heat transfer than would be observed in conventional lyophilization and was postulated to cause the sublimation front to curve. We therefore present a 2D level set (LS) model, which can address some of the uncertainties of Alexeenko et al. (2025) LC model, and use its results to present a revised LC model.

A multitude of mathematical techniques are available for treating Stefan problems, or problems where a boundary position is unknown, in one coordinate dimension (Myers 2025); in the case of lyophilization, the long-standing 1D model explicitly represents front height as a single variable. In 2 or more dimensions, this kind of exact analytical treatment of a moving boundary is difficult or, usually, intractable, so numerical approaches are the standard. These fall into several categories, such as explicit representation of the boundary location (which is the simplest, except when the interface undergoes topological changes), implicit representations such as by the level set method (Shaikh et al. 2016; Osher and Fedkiw 2003), or diffuse interface models like the enthalpy-porosity approach (Voller and Prakash 1987) which do not precisely represent the boundary’s location.

In previous 2D simulations of lyophilization (Velardi and Barresi 2008; Pikal et al. 2005), as in COMSOL (2020), explicit front representations are used, which are suitable when the sublimation front deviates only a little from a planar shape. These simulations end when any part of the front reaches the bottom, a concession which simplifies the numerical implementation but prevents simulation of the end of drying. Diffuse interface models, such as the enthalpy-porosity framework used in Bobba et al. (2020), naturally extend to a situation where the front is radically curved and can reach the end of drying. However, in situ measurements such as Nakagawa et al. (2018); Palmkron et al. (2025) show a visually sharp interface; the pore-scale analysis of Thomik et al. (2022) indicates the sublimation front is diffuse, but across a width much smaller than the total size of the frozen cake, so for a vial-scale simulation the sharp interface is a good approximation. To treat a sharp front while simultaneously allowing significant front curvature, we develop an implicit representation with the level set method.

This 2D vial-scale model will also be useful in utilizing new advances in pore-scale understanding. Experimental techniques like X-ray micro-CT scanning (Parker et al. 2010; Haeuser et al. 2018) and neutron imaging (Nakagawa et al. 2018; Palmkron et al. 2023) combine with computational infrastructure equipped to process the substantial volume of data yielded by these techniques (Ma et al. 2025) to make pore structure measurements of lyophilized material more accessible. Simulation studies like Thomik et al. (2023) have begun to couple this data to heat and mass transfer behavior at the pore scale, but the exact relations between pore structure and macroscale mass transfer behavior in lyophilization are still unknown. The LS model can investigate this effect, and can also investigate imperfect thermal probe placement in a vial. It is well-known in the lyophilization community that placing a thermocouple precisely at the bottom center of the frozen product is difficult, but the consequences for observed temperature profiles are infrequently discussed in literature. Since the LS model of necessity computes temperatures throughout the dry layer, a “virtual thermocouple” in the simulation can assess the apparent temperature that would be observed for a thermocouple displaced from the bottom center.

This work is structured as follows. In Level set model for lyophilization simulation, the 2D level set (LS) model is introduced; in Numerical implementation of level set model its numerical treatment is detailed. In Electromagnetic simulation we introduce an electromagnetic simulation setup, intended to orient physical intuition without being quantitatively exact or scalable. The experimental cases we use for model validation are described in Experimental cases and summarized in Table 4. We show electromagnetic simulation results, which provide order-of-magnitude estimates for two empirical microwave heating parameters, in Electric field strength and parameters. Results with the LS model are presented in Level set model results, both comparing to an experiment and showing a hypothetical case. Based on the LS model results, in Methods - updating simplified model we recommend improvements to the 0D lumped capacitance (LC) model of Alexeenko et al. (2025). We give the details of the parameter fitting for the LC model in Parameter fitting for lumped capacitance model and list all empirical parameters (including for comparable literature models) in Table 6. In Results and discussion - updated simplified model we compare versions of the LC model, examine the revised model’s behavior across some experimental cases from Alexeenko et al. (2025) and others from Bhambhani et al. (2021) and Gitter et al. (2019), including one comparison to the model of Park et al. (2021). In the appendix, we show model-experiment temperature comparisons for all available cases not examined elsewhere.

## Methods - simulation and experiments

### Level set model for lyophilization simulation

**Fig. 1:**
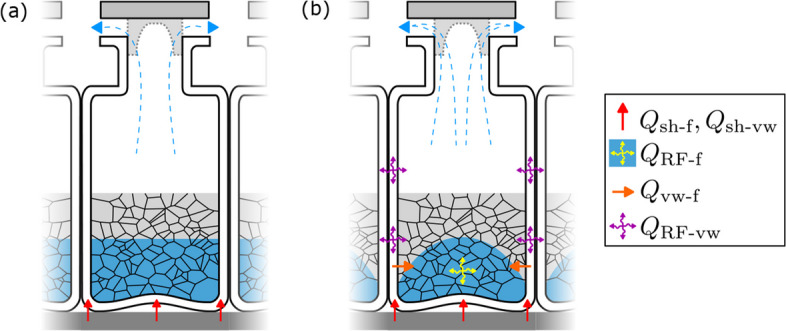
Schematic indicating distinctions in the heat transfer during primary drying between (**a**) conventional and (**b**) microwave-assisted lyophilization. Frozen product and dried “cake” regions are indicated in blue and gray, respectively

**Fig. 2:**
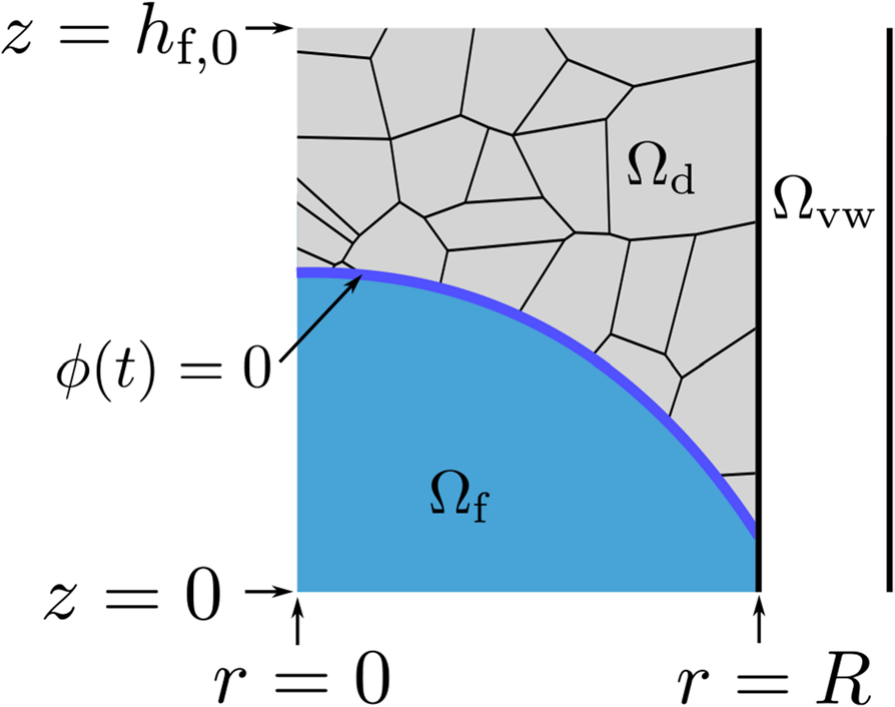
Axisymmetric simulation domain for the level set (LS) model, with the sublimation front represented by a level set function $\phi$

In the following development, we use the following subscripts: $\mathrm{vw}$ for the outer vial wall, $\mathrm{f}$ for the frozen layer of the product during drying, $\mathrm{d}$ for the dry layer, and $\mathrm{sh}$ for the shelf. A schematic of the heat transfer phenomena is shown in Fig. 1, which differs from Alexeenko et al. (2025)’s Fig. 2 only in that the shelf-to-vial-wall heating is not shown separately from shelf-to-product heating.

For this detailed simulation, we treat three domains: the frozen product, the dried cake, and the vial wall. This is schematically illustrated in Fig. 2. We assume that

1. Vapor flow does not lead to convective heat transfer in the dry layer ($\frac{\rho _{\mathrm{vap}} C_{\mathrm{p,vap}}}{\rho _{\mathrm{solid}}C_{\mathrm{p,solid}}} \ll 1$).
2. The gas in the vial headspace and pore space is essentially pure water vapor (Yoon and Narsimhan 2023).
3. The frozen product temperature is pseudosteady and does not vary vertically, but varies radially (see Vertical temperature variation).
4. The dried product temperature is pseudosteady, but varies both radially and vertically.
5. The temperature of the vial wall is approximately uniform, at least across the height of the drug product fill.
6. The system is radially symmetric, since a typical vial is surrounded by other identical vials on the shelf.

In conventional freeze drying, heat transfer through the dry layer is often not treated directly (see Velardi and Barresi (2008), section 6.1). However, since the vial wall plays a more significant role in microwave-assisted freeze drying, we separately solve energy balances in the frozen layer, dry layer, and vial wall. We also solve a mass balance for the mass transfer of water vapor in the dried region. The quantities we must solve for can be summarized as follows:

1. Sublimation front position as a function of time (by level set $\phi (r,z,t)$)
2. Pseudosteady frozen layer temperature $T_{\mathrm{f}}(r, t)$
3. Pseudosteady dry layer temperature $T_{\mathrm{d}}(r,z,t)$
4. Lumped vial wall temperature $T_{\mathrm{vw}}(t)$
5. Pseudosteady water vapor partial pressure in dry layer $p(r,z,t)$

To represent the interface, we define a level set function $\phi (r,z, t)$, which is defined everywhere inside the vial. This function is set to 0 on the sublimation front, taking positive values on the dry portion of the domain and negative values in the frozen domain. We can thus mathematically define the frozen domain by $\Omega _{\mathrm{f}} = \{(r,z) : \phi (r,z) < 0\}$ and the dried domain by $\Omega _{\mathrm{d}} = \{(r,z) : \phi (r,z)> 0 \}$.

In the dried region $\Omega _{\mathrm{d}}$, we solve a pseudosteady conduction equation for the temperature $T_{\mathrm{d}}$:

1 $$\begin{aligned} k_{\mathrm{d}} \nabla ^2 T_{\mathrm{d}} + Q'''_{\mathrm {RF-d}} = k_{\mathrm{d}} \left[ \frac{1}{r} \frac{\partial }{\partial {r}} \left( r \frac{\partial {T}_{\mathrm{d}}}{\partial {r}} \right) + \frac{\partial ^2T_{\mathrm{d}}}{\partial z^2} \right] + Q'''_{\mathrm {RF-d}} = 0 \end{aligned}$$

where $k_{\mathrm{d}}$ is the approximate thermal conductivity of the porous matrix and $Q'''_{\mathrm {RF-d}}$ is microwave heating of the dry layer, which for simplicity we take to be zero but is nonetheless included in the model implementation.

In the frozen region, we assume the temperature does not vary in the vertical direction (the Biot number, given below in Table 3, is small). However, since equilibrium vapor pressure depends very strongly on temperature, we allow radial temperature variation. We thus solve a pseudosteady conduction equation spatially averaged over the *z* direction (the “fin approximation”) for $T_{\mathrm{f}}$ in $\Omega _{\mathrm{f}}$:

2 $$\begin{aligned} 0 = k_{\mathrm{f}} \frac{1}{r} \frac{\partial }{\partial {r}} \left( r \frac{\partial \bar{T_{\mathrm{f}}}}{\partial {r}} \right) + \frac{K_{\mathrm {sh-f}} ( T_{\mathrm{sh}} - \bar{T_{\mathrm{f}}} ) + Q''_{\mathrm{front}}}{h_{\mathrm{f}}(r)} + Q'''_{\mathrm {RF-f}} \end{aligned}$$

where $K_{\mathrm {sh-f}}$ is the shelf-to-frozen-layer heat transfer coefficient, generally denoted $K_v$ in literature, and $k_{\mathrm{f}}$ is the thermal conductivity of the frozen layer, taken to be that of ice. $Q'''_{\mathrm {RF-f}}$ is volumetric heating of the frozen layer, evaluated according to Eq. (10), and $Q''_{\mathrm{front}}$ is an energy balance at the front accounting for both heat conduction at the sublimation front and the latent heat of sublimating ice:

3 $$\begin{aligned} Q''_{\mathrm{front}} = \frac{1}{\partial \phi /\partial z} \left( -k_{\mathrm{f}} \frac{\partial \phi }{\partial {r}} \frac{\partial \bar{T_{\mathrm{f}}}}{\partial {r}} + k_{\mathrm{d}} \vec {n} \cdot \nabla T_{\mathrm{d}} + \Delta H_{\mathrm{sub}} \dot{m}'' \right) . \end{aligned}$$

The local mass flow at the interface $\dot{m}'' = b\,\vec {n}\cdot \nabla p$ is evaluated by solving for the pseudosteady water vapor pressure *p* throughout the dried domain $\Omega _{\mathrm{d}}$:

4 $$\begin{aligned} \nabla \cdot (b\nabla p) = \frac{1}{r}\frac{\partial }{r} \left( br\frac{\partial {p}}{\partial {r}} \right) + \frac{\partial }{\partial {z}}\left( b\frac{\partial {p}}{\partial {z}}\right) = 0 . \end{aligned}$$

The local mass transfer coefficient *b* (akin to a permeability, but with units of time) is defined from the “dusty gas” model (Mason and Malinauskas 1983; Velardi and Barresi 2008), where we assume flow of essentially pure water vapor through a porous medium:

5 $$\begin{aligned} b \equiv \frac{M_w}{R_uT} \left( l\sqrt{\frac{R_uT}{M_w}} + \frac{\kappa }{\mu }p \right) \approx l\sqrt{\frac{M_w}{R_uT}}; \quad [=] \quad s \end{aligned}$$

$M_w$ here is the molar mass and $R_u$ the universal gas constant, with *T* taken as the local dry layer temperature $T_{\mathrm{d}}$. The two dusty gas parameters *l* and $\kappa$ have units of length and area, respectively, and loosely correspond to rarefied Knudsen diffusion and bulk viscous flow although their exact meaning is more nuanced. For the current work, we set $\kappa$ to zero although the model implementation does not require this and allows for the slight nonlinearity in Eq. (4) caused by the dependence of *b* on *p*.

With this single porous structure parameter *l*, we can account for spatial variation of pore structures by letting *l* vary spatially. To estimate *l*, we begin with the well-known mass transfer resistance of Pikal et al. (1983)

6 $$\begin{aligned} R_p=a_0 + \frac{a_1h_{\mathrm{d}}}{1+ a_2h_{\mathrm{d}}} , \end{aligned}$$

which has three empirical parameters (in other works denoted $R_0$, $A_1$, and $A_2$), which we call $a_0$, $a_1$, and $a_2$ to reduce collision of notation. In a similar fashion to Eq. 31 of Pikal et al. (2005), we can use empirically determined $a_0$, $a_1$, and $a_2$ to estimate vertical variation in the porous length scale *l*. For a purely planar front,

7 $$\begin{aligned} R_p = a_0 + \int _{h_{\mathrm{f}}}^{h_{\mathrm{f0}}} \frac{1}{b} dz; \end{aligned}$$

we then take a derivative $d/dh_{\mathrm{d}}$ and use the definition of *b* to solve for *l*:

8 $$\begin{aligned} l(z) = \sqrt{\frac{RT}{M_w}} \frac{1}{dR_p/dh_{\mathrm{d}} (z)} = \sqrt{\frac{RT}{M_w}} \frac{\left[ 1 + a_2 (h_{\mathrm{f0}} - z)\right] ^2}{a_1} . \end{aligned}$$

Here, *T* may be given a constant, representative value from experimental conditions to simplify evaluation of this weakly temperature-dependent property. Since in the 1D model all radial variation is averaged out, no such expression can be derived for how *l* might vary radially. This vertical estimation is convenient for direct comparison of model behavior when $R_p$ is known, but in principle the parameters *l* and $\kappa$ might be estimated directly from measurements of porous structures taken after drying, such as those in Ma et al. (2025).

The vial wall temperature, at least in the region where it touches the product $\Omega _{\mathrm{vw}}$, is taken to be spatially uniform but varying in time. The vial wall exchanges heat with the dried and frozen layers according to a heat transfer coefficient $K_{\mathrm {vw-f}}$,

9 $$\begin{aligned} m_{\mathrm{vial}} C_{\mathrm{p,vial}} \frac{dT_{\mathrm{vw}}}{dt} = V_{\mathrm{vial}}Q'''_{\mathrm {RF-vw}} + \int _{\mathrm{0}}^{h_{\mathrm{f0}}} K_{\mathrm {vw-f}} (T_{\mathrm{vw}} - T_{\mathrm {f/d}}) 2\pi R\,dz + (A_v-A_p)K_{\mathrm {sh-f}}(T_{\mathrm{sh}}-T_{\mathrm{vw}}) . \end{aligned}$$

The term $(T_{\mathrm{vw}}-T_{\mathrm {f/d}})$ is a local temperature difference between vial wall and either dried or frozen layer, which is then integrated across the radial product-vial boundary having an area $2\pi Rh_{\mathrm{f0}}$. The vial-wall-to-frozen-layer coefficient $K_{\mathrm {vw-f}}$ would be infinite if the product and glass temperature matched at the boundary (i.e., no thermal contact resistance). In practice, experimental data (as shown below) suggest that the vial takes very different temperatures from the frozen product, and our experimental fits below suggest that $K_{\mathrm {vw-f}}$ is of a similar order of magnitude to the shelf-to-frozen-layer heat transfer coefficient $K_{\mathrm {sh-f}}$.

The volumetric heating in the frozen layer $Q'''_{\mathrm {RF-f}}$ and in the vial wall $Q'''_{\mathrm {RF-vw}}$ is evaluated according to

10 $$\begin{aligned} Q'''_{\mathrm {RF-j}} = 2\pi \epsilon _{\mathrm{0}} \epsilon _j'' f|E|^2; \quad |E|^2 = \left( \frac{P_{\mathrm{in}}(t)}{N_{\mathrm{vials}}}B_j \right) , j \in \mathrm{vw, f, d}; \end{aligned}$$

with $\epsilon _0$ the vacuum permittivity, *f* the microwave field frequency, $\epsilon ''_f$ and $\epsilon ''_{\mathrm{vw}}$ material-specific dielectric losses, $P_{\mathrm{in}}$ the nominal microwave input power, and $B_{\mathrm{f}}$ and $B_{\mathrm{vw}}$ as empirical fit parameters with an estimated magnitude given in Electric field strength and parameters. Witkiewicz and Nastaj (2010) give this equation without the factor of 2, but we follow Abdelraheem et al. (2022) and Meredith (1998) and include the 2.

The sublimation front moves according to a transport equation

11 $$\begin{aligned} \frac{\partial \phi }{\partial {t}} + \vec {v}_{\mathrm{int}} \cdot \nabla \phi = 0, \end{aligned}$$

where the interface velocity $\vec {v}_{\mathrm{int}}$ is computed by a mass balance about the sublimation front

12 $$\begin{aligned} \vec {v}_{\mathrm{int}} = -\vec {n} \frac{\dot{m}''}{\varphi \rho _{\mathrm{ice}}} \end{aligned}$$

with $\dot{m}'' = b\,\vec {n} \cdot \nabla p$ local mass flux due to sublimation, identical to that in Eq. (3); $\vec {n} = \nabla \phi$ is the interface normal, and $\varphi$ is the porosity of the dried layer, estimated as $(\rho _{\mathrm{water}}-c_{\mathrm{solids}})/(\rho _{\mathrm{water}})$ with $c_{\mathrm{solids}}$ the formulation solids content in units of mass per liquid volume. This velocity is computed along the interface, then extrapolated throughout the domain by a procedure known as “velocity extension" Chopp (2009) in order to solve Eq. (11).

The other boundary conditions necessary to close the system of equations are as follows. At the sublimation front, the pressure is dictated by equilibrium vapor pressure over ice and the temperatures match:

13 $$\begin{aligned} p= & p_{\mathrm{sub}}(T_{\mathrm{f}}) , \quad p_{\mathrm{sub}} = p_{\mathrm{ref}}\mathrm{exp}\left( \frac{\Delta H_{\mathrm{sub}}M_w}{R_uT_{\mathrm{f}}(r)}\right) \end{aligned}$$

14 $$\begin{aligned} T_{\mathrm{d}}= & T_{\mathrm{f}} \end{aligned}$$

Note also that Eq. (12) is a mass balance at the front, and Eq. (3) is an energy balance at the front.

Boundary conditions for the temperature at the top of the cake, bottom of the vial, radial vial wall, and vial axis of symmetry are applied identically to both the frozen and dried domains. These external boundary conditions are expressed fully in Table 1. They are largely zero-flux (homogeneous Neumann) conditions, with the exception of energy balances at the vial sides and bottom and the mass balance on top of the product, each of which is an inhomogeneous Robin boundary condition. Since these external boundary conditions are all of either Neumann or Robin type, the system could have infinitely many solutions differing by a constant, if these were all the boundary conditions in the problem. However, the boundary conditions applied at the sublimation front eliminate this concern, especially the equilibrium vapor pressure relation which couples temperatures and pressures.

**Table 1:** External boundary conditions used for 2D level set model. Note that the zero-thickness mass transfer resistance $a_0$ is the same empirical parameter as in Eq. (6)

Condition	Location	Description
$\frac{\partial {T}}{\partial {r}} = 0$	$r= 0$	axisymmetric
$-k\frac{\partial {T}}{\partial {r}} = K_{\mathrm {vw-f}} (T - T_{\mathrm{vw}})$	$r= R$	exchange with wall
$k\frac{\partial {T}}{\partial {z}} = K_{\mathrm {sh-f}}(T - T_{\mathrm{sh}})$	$z= 0$	exchange with shelf
$\frac{\partial {T}}{\partial {z}} = 0$	$z= h_{\mathrm{f,0}}$	no heat flux out top
$\frac{\partial {p}}{\partial {r}} = 0$	$r= 0$	axisymmetric
$\frac{\partial {p}}{\partial {r}} = 0$	$r= R$	impermeable vial wall
$\frac{\partial {p}}{\partial {z}} = 0$,	$z= 0$	impermeable vial bottom
$-b\frac{\partial {p}}{\partial {z}} = \frac{p-p_{\mathrm{ch}}}{a_0}$	$z= h_{\mathrm{f,0}}$	zero-thickness mass transfer resistance

**Table 2:** Summary of equations solved in level set model

Eq.	Short-form equation	Domain	Physical meaning
(1)	$k_{\mathrm{d}} \nabla ^2 T_{\mathrm{d}} = 0$	Dry layer	Energy balance
(2)	$k_{\mathrm{f}} \nabla ^2_r T_{\mathrm{f}} + (K_{\mathrm {sh-f}}\Delta T + Q''_{\mathrm{front}})/h_{\mathrm{f}}(r) = 0$	Frozen layer	Energy balance
(3)	$Q''_{\mathrm{front}} = \frac{1}{\partial \phi /\partial z} \left( -k_{\mathrm{f}} \frac{\partial \phi }{\partial {r}} \frac{\partial \bar{T_{\mathrm{f}}}}{\partial {r}} + k_{\mathrm{d}} \vec {n} \cdot \nabla T_{\mathrm{d}} + \Delta H_{\mathrm{sub}} \dot{m}'' \right)$	Sublimation front	Energy balance
(4)	$\nabla \cdot (b \nabla p) = 0$	Dry layer	Mass balance
(9)	$mC_p \frac{dT_{\mathrm{vw}}}{dt} = VQ'''_{\mathrm {RF-vw}} + K_{\mathrm {vw-f}}\Delta T A_{\mathrm{side}} + (A_v-A_p)K_{\mathrm {sh-f}}\Delta T$	Vial wall	Energy balance
(11)	$\frac{\partial \phi }{\partial {t}} + \vec {v}_{\mathrm{int}} \cdot \nabla \phi = 0$	Dry & frozen layers	Front motion
(12)	$\vec {v}_{\mathrm{int}} = -\vec {n} \frac{\dot{m}''}{\varphi \rho _{\mathrm{ice}}}$	Sublimation front	Mass balance

A summary of the empirical fit parameters used in this model is given below in Table 6, and the governing equations for the level set model are summarized in Table 2.

#### Vertical temperature variation

The validity of assuming an axially uniform temperature in the frozen layer and the vial wall can be assessed by considering the Biot number, which is defined as $\mathrm{Bi}=\frac{KL}{k}$ for *K* an external heat transfer coefficient, *L* a length scale, and *k* an internal thermal conductivity. For small values of the Biot number, e.g. $\mathrm{Bi} < 0.1$, the spatial variation in temperature is negligible, often called the “lumped capacitance” approximation. Typical values of the Biot number for primary drying are given in Table 3. The lumped capacitance assumption is not used in typical models for primary drying, such as Velardi and Barresi (2008) and Pikal et al. (2005), where instead the vertical heat transfer is assumed to be pseudosteady, yielding a simple, linear analytical solution for temperature in the frozen region. Typical temperature differences for these models are on the order of 0.5K to 2 K, such as seen in Velardi and Barresi (2008)’s Fig. 9. However, no such simple analytical result is available when radial heat transfer must be considered, especially for a nonplanar sublimation front. Since $\mathrm{Bi}$ is likely to be larger for the radial direction than the vertical direction, we focus here on the radial variation and use the lumped capacitance approximation in the vertical direction.

$\mathrm{Bi}$ may exceed 0.1 vertically for products with a large fill depth, i.e. greater than 1 cm, if the product is also dried at high pressures so that the heat transfer coefficient is large. Neglecting vertical temperature gradients in this regime will slightly overestimate heat transfer rate (by increasing $T_{\mathrm{sh}}-T_{\mathrm{f}}$) and drying rate (by increasing $p_{\mathrm{sub}}(T_{\mathrm{sub}})-p_{\mathrm{ch}}$), thus underestimating total drying time. However, this regime will often be avoided for other practical reasons: large fill depths increase drying time in general, both by increasing the ratio of volume to surface area and by increasing the mass transfer resistance; the combination of a large mass transfer resistance and large heat transfer coefficient will also lead to high product temperatures.

For all the experiments considered here, the Biot number in the ice (in vertical and radial directions) is given in Table 4. $\mathrm{Bi}_{\mathrm{f,vert}}$ does not exceed 0.1 for any of these cases, and $\mathrm{Bi}_{\mathrm{f,rad}}$ exceeds 0.1 only in cases M3 (Alexeenko et al. 2025); S2 (Bhambhani et al. 2021); F1, F2, and F4 (Gitter et al. 2019).

**Table 3:** Expected ranges of the Biot number for primary drying in a vial, across different directions and domains. It is reasonable to assume a spatially uniform temperature when $\mathrm{Bi}<0.1$

Direction	Region	Material	*k*,$\mathrm{Wm}^{-1} \mathrm{K}^{-1}$	Heat transfer coefficient	*K*,$\mathrm{Wm}^{-2} \mathrm{K}^{-1}$	Length scale, cm	$\mathrm{Bi}$
Vertical	Frozen layer	Ice	2.4^1^	$K_{\mathrm {sh-f}}$	5 to 25	0.1 to 2	0.004 to 0.2
Vertical	Vial wall	Glass	1.0^2^	$K_{\mathrm {sh-f}}$	5 to 25	0.1 to 2	.01 to 0.5
Radial	Frozen layer	Ice	2.4	$K_{\mathrm {vw-f}}$	5 to 150	0.7 to 1.5	0.01 to 0.9
Radial	Vial wall	Glass	1.0^2^	$K_{\mathrm {vw-f}}$	5 to 150	0.1	0.005 to 0.06
Radial	Dry layer	Cake	0.007^3^	$K_{\mathrm {vw-f}}$	5 to 150	0.7 to 1.5	5 to 130

^1^From Slack (1980), at 250 K

^2^From Bansal and Doremus (1986), for borosilicate glass

^3^Estimated as (Maccarthy and Fabre 1989) times a typical volume fraction of solids in the dry layer, e.g. 0.05 for a 5% w/v solution

### Numerical implementation of level set model

Although inspiration was taken from Shaikh et al. (2016) in choosing the level set method, the discretization described there is not used here. Following Osher and Fedkiw (2003); Gibou et al. (2002); Gibou and Fedkiw (2005), a second order finite difference method is used to discretize the spatial derivatives of the partial differential equations for the 2D model. At grid points near the sublimation front, the finite difference discretization takes into account the position of the sublimation front, which is identified by linear interpolation of the level set field $\phi (r,z,t)$ following Min and Gibou (2008). The level set field must also be “reinitialized” periodically, a known and well-discussed aspect of the level set method (Osher and Fedkiw 2003), and the approach used for this is the HCR2 algorithm from Hartmann et al. (2010). To compute volumes and surface areas, discrete Heaviside and delta functions are used, as described in Min and Gibou (2007).

The heat and mass transfer in both the dry and frozen domains are taken to be pseudosteady, so at a given time step, temperatures and pressures are computed based on the current position of the sublimation front and temperature of the vial wall. For given values of the frozen temperature $\bar{T}_{\mathrm{f}}$, the dry layer temperatures $T_{\mathrm{d}}$ and water vapor pressures *p* can be computed. However, due to the Clausius-Clapeyron relationship governing sublimation pressure as a function of temperature, $\bar{T}_{\mathrm{f}}$ has a nonlinear dependence on *p*. This yields, formally, a differential-algebraic equation (DAE) system, where the level set function $\phi (r,z,t)$ and vial wall temperature $T_{\mathrm{vw}}(t)$ are differential variables and the discretized governing equation of $\bar{T}_{\mathrm{f}}(r)$ is a set of algebraic constraints.

This model is implemented in Julia (Bezanson et al. 2017), a programming language designed for use in scientific computing, with good features for reproducibility, reuse of code, and tools for managing a scientific code base (e.g. DrWatson.jl (Datseris et al. 2020), which is utilized). No existing implementation of the level set method was found, so spatial discretizations are written from scratch. However, where possible, the SciML ecosystem is leveraged, which was used for solution of sparse linear systems (LinearSolve.jl), nonlinear systems (NonlinearSolve.jl), and time integration (OrdinaryDiffEq.jl (Rackauckas and Nie 2017)). For linear systems, the SparspakFactorization() algorithm is used. The nonlinear DAE system can be integrated in time with the FBDF() algorithm, with the exception that a finite-time instability is encountered when the sublimation front detaches from the wall. After this point, the algebraic equations are solved by the NewtonRaphson() algorithm at each time point with explicit and adaptive time integration using the SSPRK43() scheme (Kraaijevanger 1991; Conde et al. 2022; Ranocha et al. 2022).

On the author’s laptop with an 11th Gen Intel Core i7–1165G7 CPU, this implementation of the LS model typically runs in 1–10 minutes (with the highest-resolution case below taking about an hour); in contrast, the lumped capacitance models below take around a millisecond, and traditional CFD simulations may take much longer.

**Fig. 3:**
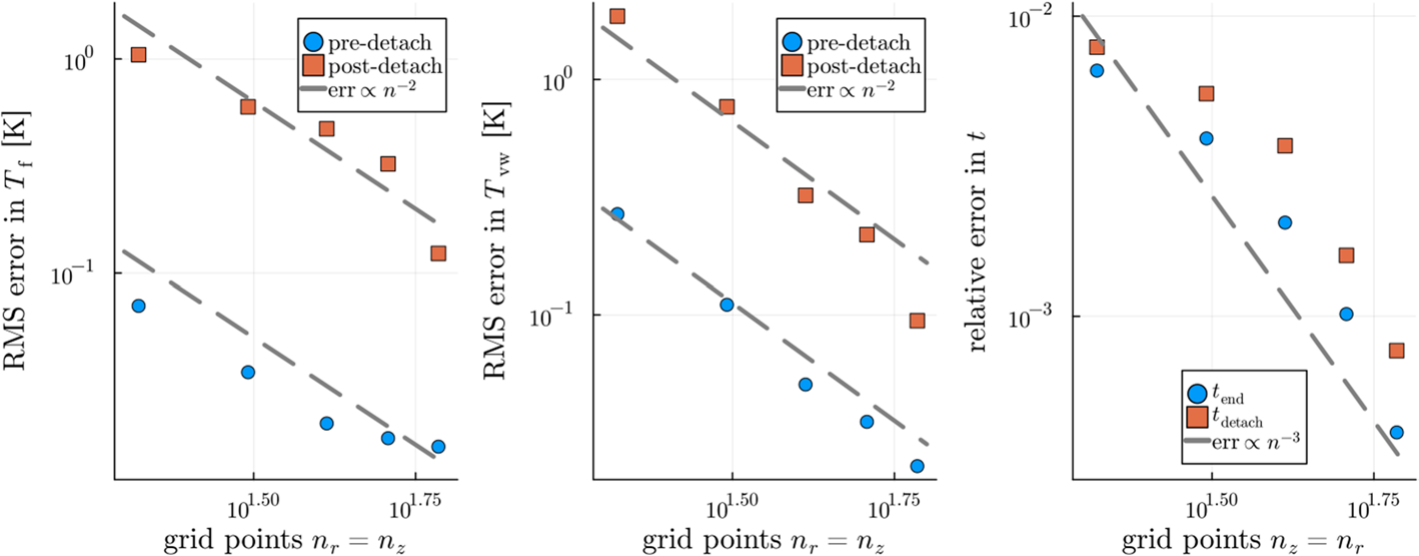
RMS error in the bottom-center frozen temperature, vial wall temperature, and total drying time as a function of number of grid points, in the *r* and *z* directions respectively

In Fig. 3, we analyze the convergence of model-predicted frozen temperature (located at the bottom center), vial wall temperature, and total primary drying time when compared in an $L_2$ sense to a high-resolution simulation. The temperatures are compared pre- and post-detachment, detachment referring to the point in time when the sublimation front detaches from the wall and ice no longer covers the full radial extent of the product. The outer radial boundary condition on the frozen temperature $T_{\mathrm{f}}$ changes at that point, and therefore so does the discretization. For a number of grid points *n*, identical in the radial and axial directions, the error in both temperatures goes as $O(n^{-2})$. The error in time to detachment and total drying time both decrease with $O(n^{-3})$. This confirms that the discretizations of temperature and pressure are second order, and that the WENO discretization used for treating the level set field is at least third order.

The implementation of the LS model is available as open source software, termed LevelSetSublimation.jl, with the MIT license (Wheeler 2026a). The implementation of the LC model is contained in the package LyoPronto.jl, which is available from the General package registry for Julia (Wheeler et al. 2026).

### Electromagnetic simulation

**Fig. 4:**
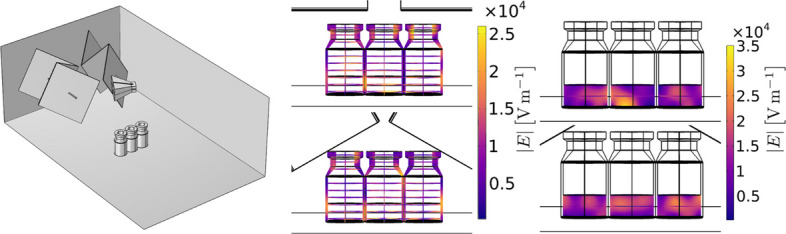
Simulation geometry, simulated electric field strength |*E*| in glass vials, and simulated field strength in ice. The horn antenna, suspended from the resonant cavity’s top, is pointed obliquely at the electromagnetic stirring mechanism (Alexeenko et al. 2025), and approximate models of three 6 mL vials (which are 4 cm tall and contain 3.14 mL of ice) are included. The two shown realizations are at two different angles of the electromagnetic stirring mechanism ($\theta = 0^{\circ }\ \& \ 240^{\circ }$)

To provide basic insight into the electric field behavior in vials containing frozen material, we run full-wave electromagnetic simulations in COMSOL. The microwave chamber and electromagnetic stirrer geometry is as shown in Fig. 3 of Alexeenko et al. (2025). Here, we add approximate CAD models of 3 6R glass vials containing 3.14mL of ice (having a height of 1 cm) to the simulation geometry, with constant dielectric properties of $\epsilon '=3.13$ and $\tan \delta =\epsilon ''/\epsilon '={0.008}$ for ice (Matsuoka et al. 1996) and $\epsilon '=6.1$ and $\tan \delta =0.004$ for borosilicate glass (SCHOTT 2022). This simulation geometry is shown below in Fig. 4. A nominal power of 40 W at 9 GHz is input to the RF antenna, and the electric field is simulated throughout the cavity.

Simulations were performed in *COMSOL Multiphysics-v 5.6* (RF Module) using a frequency‑domain study with *lumped port excitation* using 40 W (9 GHz) at the input of the antenna, *impedance* boundary conditions on all external metallic structures, and *transition* boundary conditions on all internal metallic structures. A tetrahedral mesh with targeted refinement at the vial shoulder and neck as well as at the ice–glass–air interfaces resolved the shortest effective wavelength in the load with at least 5 elements per $\lambda _{\mathrm{eff}}$. The final mesh contained approximately $2.9 \times 10^{6}$ volume elements ($1.9 \times 10^{7}$ degrees of freedom), with minimum element sizes of 57 $\upmu$m in the ice and 20 $\upmu$m in the glass. Runs were executed on an *Intel® Xeon®* W3-2435 up to 3.1 GHz with 8 cores and 128GB RAM, and the simulation time was 198 min. Simulation output is electric field throughout the simulation volume; representative fields in the vial wall and ice are shown below in Fig. 4.

This simulation does not attempt to fully predict the field distribution that would be observed in a true freeze-drying cycle; rather, it is intended to demonstrate typical electromagnetic behavior in the vial and frozen product. It will also provide a rough estimate of the empirical fit parameters $B_{\mathrm{f}}$ and $B_{\mathrm{vw}}$ which appear in Eq. (10).

### Experimental cases

To validate the models presented here, we reexamine experiments presented in Alexeenko et al. (2025), where aqueous solutions of mannitol and a sucrose-mannitol mixture were dried by microwave-assisted lyophilization; we reuse the labels SM1, SM2, M1, M2, and M3 to refer to these cases. Case M4 was conducted on the same apparatus with the same materials, but the microwave power was manually turned on and off at approximately half-hour intervals.

We can likewise reexamine literature examples of microwave vacuum drying in vials. In the absence of raw data files, we extract temperatures reported in Fig. 5a, b of Bhambhani et al. (2021) and Fig. 1a-c of Gitter et al. (2019) with version 4 of WebPlotDigitizer (Rohatgi 2024).

**Fig. 5:**
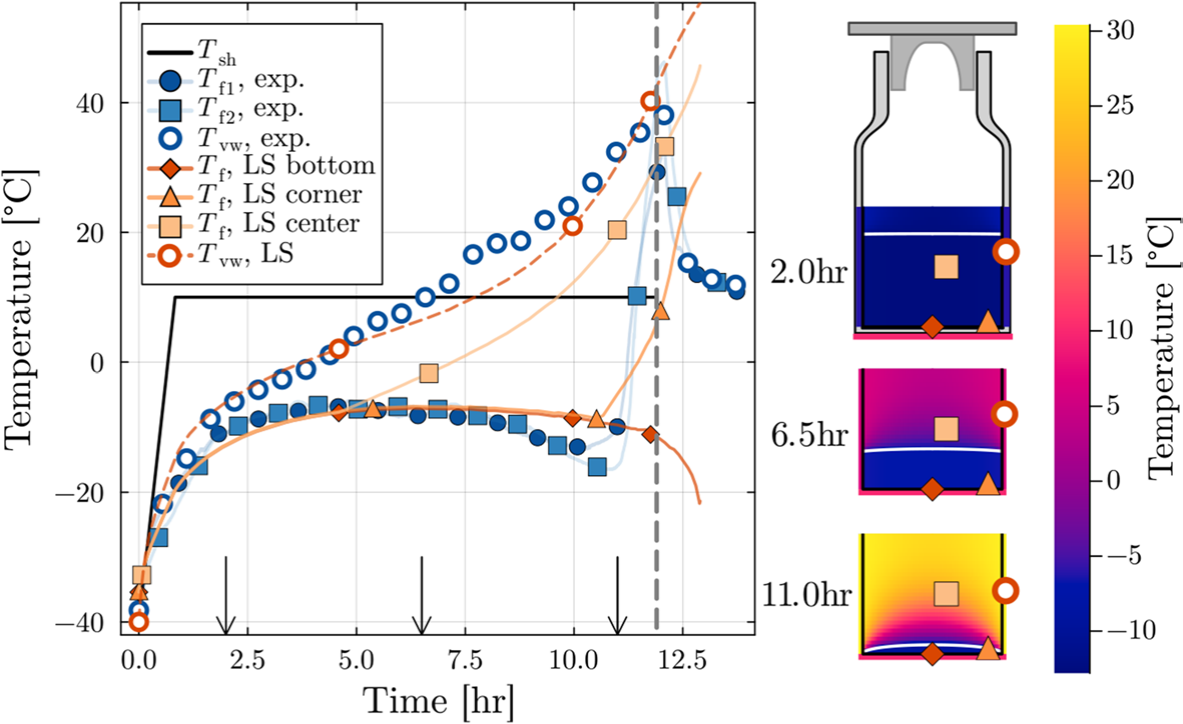
Simulation results for the level set model, compared to experimental case M1. Marked times indicate time points for which temperature fields are shown, and markers on the colored temperature fields correspond to positions of the “virtual thermocouples” for which temperatures are shown on left plot. $K_{\mathrm {vw-f}}$, $B_{\mathrm{f}}$, and $B_{\mathrm{vw}}$, like $K_{\mathrm {sh-f}}$ and $R_p$, take the same values given below in Table 7 for the lumped capacitance model, and the full simulation specification can be found in the accompanying data repository (Wheeler 2026b)

The experimental conditions for all of these cases are summarized in Table 4. Cases S1 and S2 correspond to Fig. 5a and b, respectively, of Bhambhani et al. (2021). Microwave power for case S2 is given in Table IV of Bhambhani et al. (2021). Cases F1, F2, and F4 correspond to Fig. 1a, b, and c respectively of Gitter et al. (2019). The reported pressures there are measured with a Pirani gauge which is by nature sensitive to gas composition. However, in the absence of any other measurements, these Pirani pressure measurements are used in simulation. A total count of vials in the microwave drying cycles is not given, so power cannot be compared on a per-vial basis as is done for the rest of these examples.

**Table 4:** Experimental cases considered. $\mathrm{Bi}_{\mathrm{f,vert}}$ and $\mathrm{Bi}_{\mathrm{f,rad}}$ are the Biot number in the frozen product in the vertical and radial directions, discussed in Vertical temperature variation. Heat transfer coefficients for S1, S2, F1, F2, and F4 are given in the appendix in Table 8

Case	Microwave power	Microwave frequency	Vacuum setting	Shelf temp.	Fill volume	$\mathrm{Bi}_{\mathrm{f,vert}}$	$\mathrm{Bi}_{\mathrm{f,rad}}$
	(W)	(GHz)	(mTorr)	($^{\circ }$C)	(mL)		
Formulation SM: 2% w/v sucrose + 4% w/v mannitol, container: 6R vial, load: 30 vials (Alexeenko et al. 2025)							
SM1	0	8	70	10	3	0.06	
SM2	25	8	70	10	3	0.06	0.02
Formulation M: 5% w/v mannitol, container: 6R vial, load: 17 vials, Alexeenko et al. (2025)							
M1	10	8	100	10	5	0.1	0.07
M2	10	8	100	10	3	0.06	0.07
M3	23.9^1^	18	100	−15	3	0.06	0.6
M4	15.25^1^	18	100	−15	3	0.06	0.07
Formulation S: 5% w/v sucrose, container: 3mL vial, load: 152 vials, Bhambhani et al. (2021) Fig. 5							
S1	0	2.45	40	^2^	0.7	0.01	
S2	800^1^	2.45	60	^2^	0.7	0.1	0.15
Formulations F1, F2, F4: mAb-containing, container: 10R vial, load: unknown, Gitter et al. (2019) Fig. 1							
F1	39^1^	2.45	6 to 23	^2^	2.3	0.0002	0.32
F2	58^1^	2.45	6 to 23	^2^	2.3	0.0001	0.16
F4	83^1^	2.45	4 to 15	^2^	2.3	0.01	0.18

^1^Time-averaged output power

^2^No shelves with controlled temperature, so an ambient temperature of 10$^{\circ }$C is used in its place

## Results and discussion - simulation

### Electric field strength and parameters

Figure 4 shows the simulated electric field strength for the simulation described in Electromagnetic simulation. Although both electric fields vary across the product and the vial and would produce local hot spots, these local peaks are in different places for the two different stirrer angles. When the electromagnetic stirring mechanism is rotated at tens of revolutions per minute, the local electric field peaks, which take nanoseconds to reach an electrical steady state, will move around and yield a *statistically* uniform field as shown in Alexeenko et al. (2025)’s Fig. 4.

The penetration depth, defined as the depth at which (in a 1D planar geometry) the electric heating drops to 1/*e* its boundary value, can be estimated by Meredith (1998)’s Eq. 2.10:

15 $$\begin{aligned} D_p = \frac{\lambda _0 \sqrt{\varepsilon '}}{2\pi \varepsilon ''} \end{aligned}$$

where $\lambda _0$ is the electromagnetic wavelength, $\varepsilon '$ is the real part of permittivity, and $\varepsilon ''$ is the imaginary part of permittivity (or dielectric loss coefficient). For pure ice at 18 GHz and 240 K, $D_p=1.2\ \mathrm{m}$, so complete penetration is expected. To estimate a threshold of $\varepsilon ''$ above which heating will take place mostly near the exterior of the frozen product, consider a minimum wavelength of $1.67\ \mathrm{cm}$ corresponding to 18 GHz, $\varepsilon '$ for ice of 3.15 (Matsuoka et al. 1996), and $D_p=1.5\ \mathrm{cm}$ (the radius of an especially large vial): this corresponds to $\varepsilon ''={0.3}$, much larger than that of pure ice as measured by Matsuoka et al. (1996) and larger than any measurements by Choudhary et al. (2026) for ice with concentration of sucrose up to 30% w/w.

The empirical parameters $B_{\mathrm{f}}$ and $B_{\mathrm{vw}}$, as defined in Eq. (10) and introduced by Alexeenko et al. (2025), describe the relation between electric field and nominal input power. Equivalently, they characterize 1) the overall microwave energy delivery to a product and its container and 2) the way energy is split between product and container. These parameters are therefore closely connected to the electromagnetic behavior of the lyophilizer chamber as a resonant cavity, as well as the way in which waves are scattered by the complex geometry of vials packed hexagonally on a shelf. With a randomized-field system, as used here, the overall energy delivery should be uniform in an average sense across vials. But for any given vial, the exact partitioning of energy between frozen product and container depends in a fundamental way on the field frequency, the dielectric properties of both vial wall and frozen product, and the vial geometry.

This simulation—of a small resonant cavity with 3 vials—approaches the limits of what can easily be done with commercial simulators at a reasonable computational cost. Computational cost will increase with the number of vials, as well as with the overall size of the system. Similar simulations not presented here indicate that the electric field’s distribution and strength depend strongly and nonlinearly on the exact geometry of the vial, particularly the presence of sharp corners such as at the opening and the neck of the vial. Even the overall efficiency of power delivery is dependent on the overall load size—small loads such as the three vials used in simulation may absorb less power, on a per-vial basis, than larger loads. Exact calculation of $B_{\mathrm{f}}$ and $B_{\mathrm{vw}}$ by electromagnetic simulation is therefore unattainable for even this relatively small resonant cavity, much less a full lab-scale or commercial-scale lyophilizer. Such a calculation would also rely on measurements of the dielectric properties of both glass vials and frozen product, which are not currently available. Choudhary et al. (2026), in this same special collection, begin to provide those measurements, but still not at the frequencies used here.

Nonetheless, we can use this simulation to provide a very rough estimate of the two empirical parameters. For a nominal input power of 40 W across 3 vials, the average electric field strength across the 3 vials and 3 different stirrer angles is $1.25 \times 10^{4} \mathrm{Vm}^{-1}$ in the glass and $1.4 \times 10^{4} \mathrm{Vm}^{-1}$ in the ice. Averaged across the same cases, the average microwave heat delivery is 4.6W to each vial and 0.14W to the ice. $B_{\mathrm{f}}$ and $B_{\mathrm{vw}}$ are defined as the ratio of electric field in each material to the nominal input power per vial, or $B_{\mathrm{i}} = \frac{|E|_{\mathrm{i}}^2}{P/N}$. For this simulation, then, $B_{\mathrm{f}} \approx 1.5 \times 10^{7} \Omega \mathrm{m}^{-2}$, and $B_{\mathrm{vw}} \approx 1.2 \times 10^{7} \Omega \mathrm{m}^{-2}$. But the uncertainty in dielectric parameters and strong nonlinear dependence of electric field strength on vial geometry and load size mean these estimates may still be a couple orders of magnitude different from what is experimentally observed.

Models for the lyophilization process, in the rest of this work, incorporate the temperature and frequency dependence of $\epsilon ''_{\mathrm{f}}$ using the correlation in (Matsuoka et al. 1996) for water ice, but take $\epsilon ''_{\mathrm{vw}}$ as constant with the same value for borosilicate glass as used in this simulation. The uncertainty in dielectric parameters and in electric field readily explain why $B_{\mathrm{f}}$ takes values so much larger than $B_{\mathrm{vw}}$ in Fig. 11 and Table 7.

Having provided these estimates from electromagnetic simulation, empirical estimation from temperature measurements remains the best approach for measuring $B_{\mathrm{f}}$ and $B_{\mathrm{vw}}$. We anticipate that both parameters depend on formulation, container type, fill volume, lyophilizer and microwave geometry, and microwave frequency. Fortunately, although $B_{\mathrm{f}}$ and $B_{\mathrm{vw}}$, *may* depend on all these factors, the results below in Table 7 and Appendix Table 8 suggest that these parameters may not always be so unpredictable.

### Level set model results

#### Comparison to experiment

Level set simulations agree with experimental data, as shown in Fig. 5. On the left are experimentally measured temperatures for the frozen layer in two vials and the wall in a third vial ($T_{\mathrm{f1}}$, $T_{\mathrm{f2}}$ and $T_{\mathrm{vw}}$ respectively), making 3 out of 17 vials in the batch with probes. On the right are simulated temperature distributions throughout the product at three points during primary drying. White lines on the color plots indicate the sublimation front position.

First, the model accurately fits the temperatures observed in experiment, as well as the total primary drying time as measured by Pirani and CM pressure convergence. Second, by tracking temperatures with “virtual thermocouples” in the simulation (shown with shapes on the right color fields which correspond to markers in the left graph), this model suggests that the well-known temperature rise observed near the end of primary drying occurs when a thermal probe is no longer covered in ice. After being uncovered, it approaches the temperature of the vial wall, which may be much higher than the product temperature—particularly in open-loop microwave-assisted lyophilization.

Both experimental measurements and model predictions show that temperature in the frozen layer drops near the end of drying. This can be explained from a heat transfer perspective: as the volume of the ice decreases, it absorbs less direct microwave heating, and its decreasing contact area with the vial wall reduces the total heat transfer from the vial wall. This same behavior occurs in the LC models we present below. In the LS model, we observe that frozen temperature drops precipitously once the sublimation front passes the corner of the vial at 11.5 h in Fig. 5.

#### Spatially varying pore structure

Since the model allows for spatially varying porous structure, we can use it to explore whether other physical phenomena might induce sublimation front curvature. For the experimental cases investigated here, experimental measurements are limited to temperature and so no more detailed analysis is feasible. However, this functionality could be combined with detailed measurements such as in Ma et al. (2025) to uncover more mechanistic understanding of mass transfer in lyophilization.

**Fig. 6:**
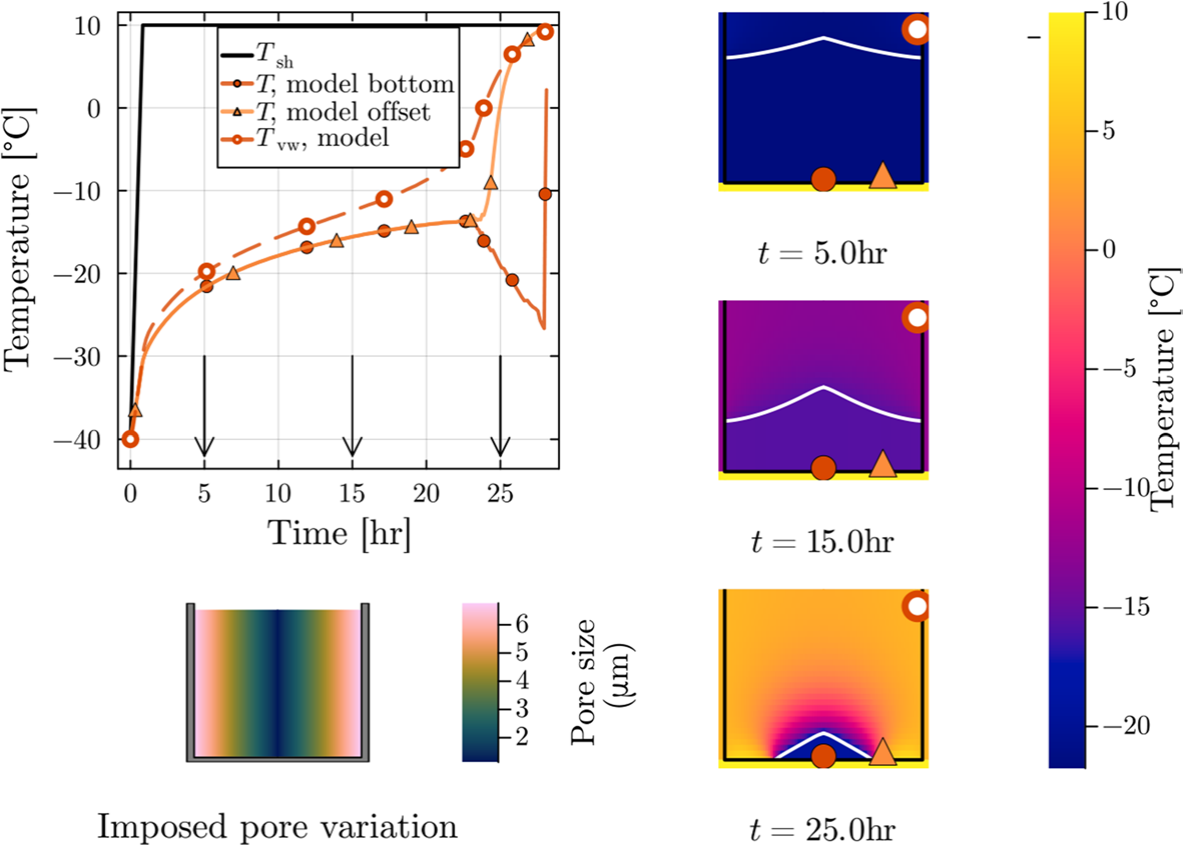
LS simulation with no microwave power and an artificial radial variation in pore size. This mass transfer effect induces much more curvature than can the radial heat transfer of microwave heating (see Fig. 5)

To demonstrate this capability, we can take the expression in Eq. (8) and add radial variation such that $l^*(r,z) = l(z)( 0.25 + 1.25r/R)$, emulating a situation where pores are 4 times smaller in the center of the product and 1.5 times larger at the outer wall. Figure 6 shows simulation results for a case closely matching case M1 (see Fig. 5), but with this pore size variation added and microwave power set to zero. The simulated interface curvature due to pore size variation is much more pronounced than curvature due to radial heat transfer. As in Fig. 5, even the thermal probe displacement in Fig. 6 (from exact center to half a radius away) causes a divergence in observed temperatures once the off-center probe is no longer in ice, with the off-center probe and vial wall both rapidly approaching shelf temperature of 10 $^{\circ }$C while the exact bottom center descends to $-28 ^{\circ }$C just before ice disappears. So long as both probe points are in the ice, measured temperatures are nearly equal, but once insulated from one another by dried material, they rapidly diverge.

#### Curvature assessment

We can estimate the effect of the front curvature on mass transfer by using the original definition of $R_p$ to compute an effective $R_p$ over time for a given level set simulation; that is,

16 $$\begin{aligned} R_{\mathrm{p,eff}}=[{p}_{\mathrm{sub}} (T_{\mathrm{f}} (r=0))-p_{\mathrm{ch}}]\cdot A_p/\dot{m}, \end{aligned}$$

for $A_p$ the inner cross sectional area of the vial and $\dot{m}$ the mass of water vapor leaving the vial. The sublimation pressure here is evaluated with the center temperature, i.e. at temperature $T_{\mathrm{f}}(r=0)$, as would be measured by an ideally-placed thermocouple in experiment. A corresponding effective dry layer height is

17 $$\begin{aligned} h_{\mathrm{d,eff}}=(1-V_{\mathrm{f}}/V_{\mathrm{fill}}) h_{\mathrm{f0}} \end{aligned}$$

with $V_{\mathrm{f}}$ the volume of frozen product. Comparing this effective resistance to the $R_p$ used with Eq. (8) to estimate *l* (our porous structure parameter), we can assess whether radial heat transfer or varying pore structure are inducing changes in $R_p$.

**Fig. 7:**
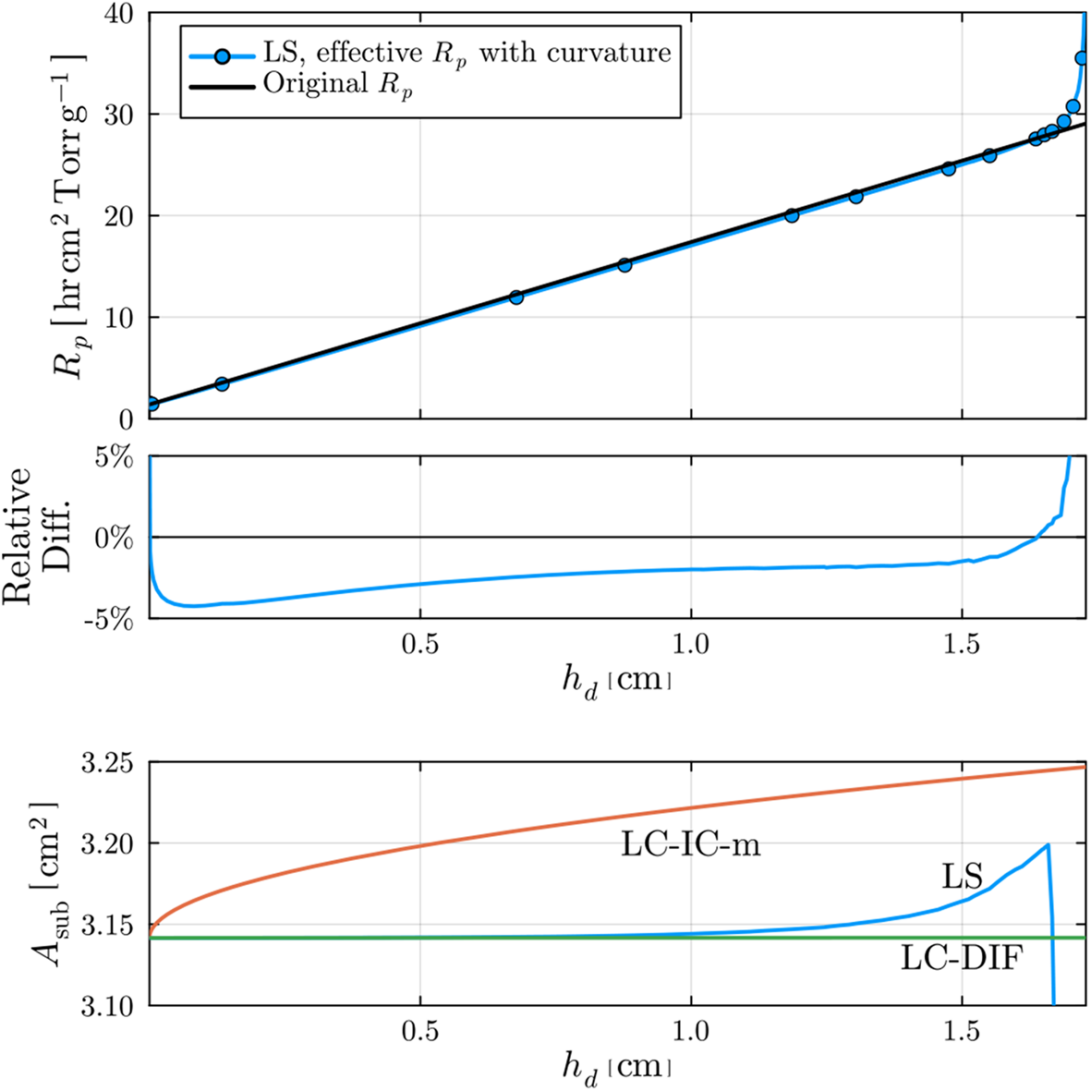
Quantification of mass transfer effects due to interface curvature for an LS simulation matched to case M1, microwave-assisted lyophilization of 5% mannitol. At top, $R_{\mathrm{p,eff}}$ from Eq. (16) is compared to the original $R_p$ used for Eq. (8). At bottom, the sublimation area is shown versus the effective dry layer height of Eq. (17), for the 2D LS model of Level set model for lyophilization simulation as well as the 0D LC-IC-m and LC-DIF models introduced in Methods - updating simplified model and shown in Fig. 10. The curvature visible in Fig. 5 induces less than a 5% difference in the effective $R_p$, which is likely within the typical uncertainty for experimental estimation of mass transfer resistance

Figure 7 shows this calculation for the same simulation shown in Fig. 5. With the exception of the very beginning and very end of drying, the effective $R_p$ is less than 4% different from the original $R_p$, a difference that would hardly be measurable in experiment. At the beginning, differences are likely due to discretization error; near the end, the minor front curvature leads to sublimation front detaching from the wall and a decreasing area for sublimation and therefore increasing the effective $R_p$. The maximum area of the sublimation front, just before it detaches from the wall, is only about 2% larger than the inner cross section of the vial. The bottom of Fig. 7 compares the sublimation area of this simulation to that of Alexeenko et al. (2025), denoted LC-IC-m in this work, and the new model termed LC-DIF (explained below in LC-IC: conduction only at ice–wall contact, curved front and LC-DIF: conduction through dry layer and ice at vial wall, flat front respectively). Although the sublimation area does change, the difference from planar behavior is small and not easily captured by a single empirical parameter.

**Fig. 8:**
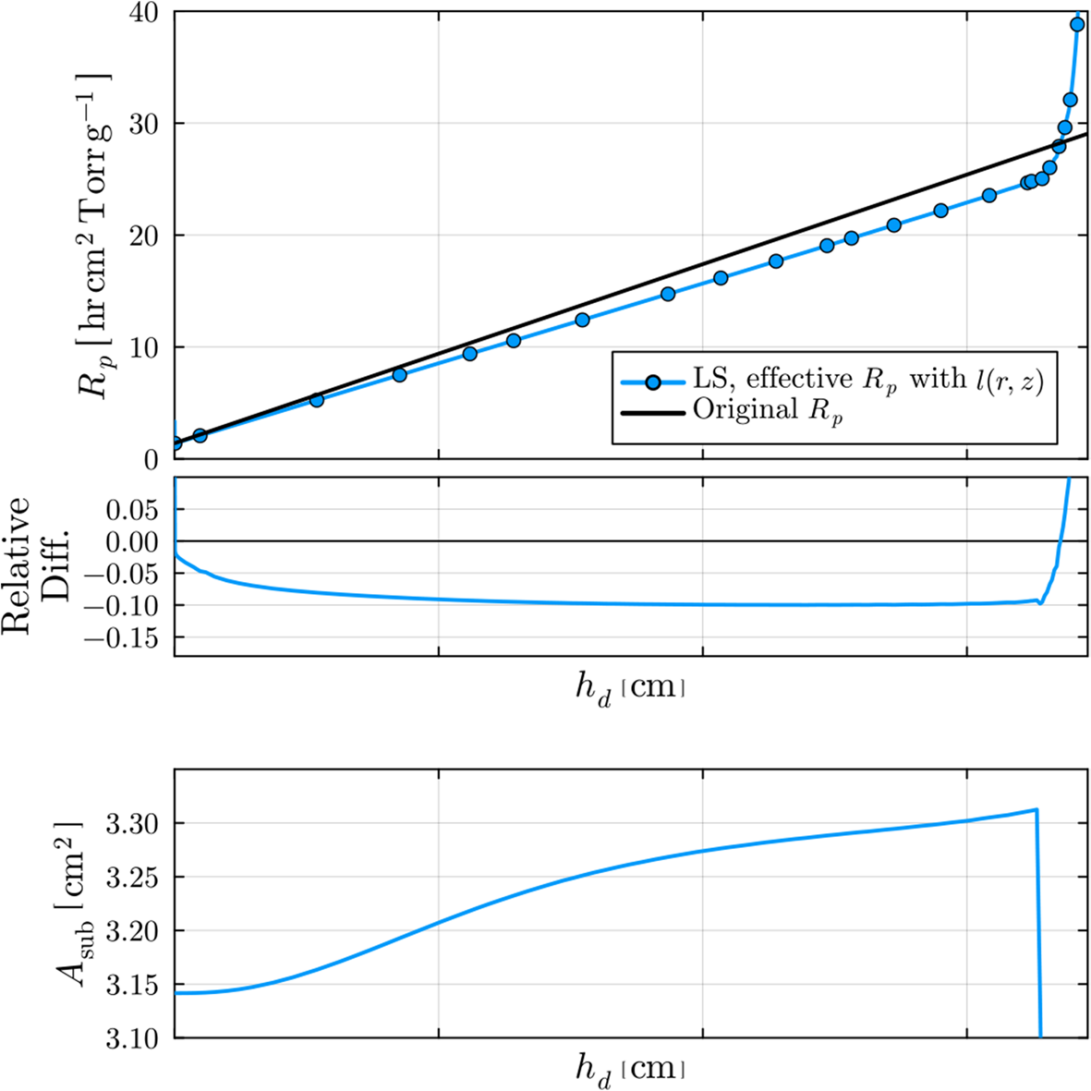
Quantification of mass transfer effects due to interface curvature, for the simulation of Fig. 6, which is similar to case M1 but with no microwave heating and artificial radial pore size variation. At top, $R_{\mathrm{p,eff}}$ from Eq. (16) is compared to the original $R_p$ used for Eq. (8). At bottom, the sublimation area is shown versus the effective dry layer height of Eq. (17). Although the interface is decidedly nonplanar, the effective $R_p$ is nearly linear, suggesting that the existing empirical model will capture and mask this sort of two-dimensional behavior

Likewise Fig. 8 shows the effective $R_p$ corresponding to Fig. 6, which does not have any microwave heating. Having 4x smaller pores at the center and 1.25x larger at the outside causes the effective $R_p$ to be about 10% smaller, which is a larger effect than the microwave heating causes in Fig. 7, but still small.

Importantly, the curve of $R_{\mathrm{p,eff}}$ vs. $h_{\mathrm{d,eff}}$ could still readily be fit by the traditional empirical form of $R_p=a_0+a_1h_{\mathrm{d}}/(1+a_2h_{\mathrm{d}})$. This means that the traditional estimation of $R_p$ for conventional lyophilization could give accurate fits to temperature, even when a strictly 1D description of mass transfer behavior is unrealistic, such as in Nakagawa et al. (2018)’s Fig. 4. Thus 0D or 1D models will be sufficient for empirically determining $R_p$ and optimizing process design as described in current best practice (Tchessalov et al. 2023). However, in future efforts to develop *a priori* predictions of $R_p$ (that is, from formulation and freezing conditions, without lyophilization cycle data), the LS model’s 2D description with spatially varying pore sizes will excel, particularly since it can run much faster than full CFD models. The functions in this model implementation (Wheeler (2026a)) for computing $R_{\mathrm{p,eff}}$ may even provide a way to directly relate porous microstructure variation to average mass transfer.

## Methods - updating simplified model

**Fig. 9:**
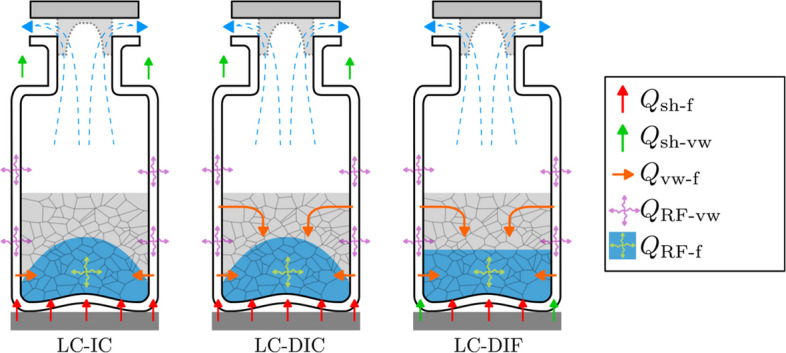
Schematic illustrating distinctions in the considered heat transfer phenomena between the LC-IC, LC-DIC, and LC-DIF models. These are not distinct experimental setups, but a difference in the mathematical model. IC means vial wall conduction is through the Ice only and front is assumed Curved; DIC means Dry layer and Ice both conduct heat from vial wall and front is Curved; DIF means Dry layer and Ice both conduct heat and front is Flat

With the benefit of the level set model, we can improve upon the lumped capacitance model originally presented in Alexeenko et al. (2025). Here we outline the model, summarize the changes in Table 5, then provide the rationale for these changes. The changes in accounted heat transfer modes are also schematically shown in Fig. 9.

The lumped capacitance approach, justified when the Biot number is small (see Table 3 and discussion in Vertical temperature variation), ignores spatial variation of temperature within the ice as well as within the vial wall. This allows the partial differential equations of the level set model to be reduced to three ordinary differential equations: one each for the temperature of the frozen layer $T_{\mathrm{f}}$, temperature of the vial wall $T_{\mathrm{vw}}$, and remaining frozen mass $m_{\mathrm{f}}$ (equivalent to the position of the sublimation front).

18 $$\begin{aligned} C_{\mathrm{p,f}} \frac{d(m_{\mathrm{f}}T_{\mathrm{f}})}{dt}= & Q_{\mathrm {RF-f}}''' V_{\mathrm{f}}(t) + Q_{\mathrm {vw-f}} + Q_{\mathrm {sh-f}} - \dot{m}_{\mathrm{flow}} \Delta H_{\mathrm{sub}} , \end{aligned}$$

19 $$\begin{aligned} C_{\mathrm{p,vial}} m_{\mathrm{vial}} \frac{d T_{\mathrm{vw}}}{dt}= & Q_{\mathrm {RF-vw}}'''V_{\mathrm{vial}} - Q_{\mathrm {vw-f}} + Q_{\mathrm {sh-vw}}, \end{aligned}$$

20 $$\begin{aligned} \frac{dm_{\mathrm{f}}}{dt}= & -\dot{m}_{\mathrm{flow}}\left( \frac{\rho _{\mathrm{solution}}}{\rho _{\mathrm{solution}}-c_{\mathrm{solid}}}\right) \end{aligned}$$

The factor of $\rho /(\rho -c)$ in Eq. (20)—where $\rho _{\mathrm{solution}}$ is the density and $c_{\mathrm{solid}}$ is the mass of solids per volume of the initial liquid solution—accounts for the mass of dried material left behind as the sublimation front moves. The model accounts for microwave heating of the vial ($Q'''_{\mathrm {RF-vw}}$) and frozen product ($Q'''_{\mathrm {RF-f}}$) as given above in Eq. (10), shelf heating of the product ($Q_{\mathrm {sh-f}}$), vial-to-frozen-product heat transfer ($Q_{\mathrm {vw-f}}$), and vial wall interaction with shelves ($Q_{\mathrm {sh-vw}}$). These interactions are schematically indicated in Figs. 1 and 9, and explained in the following sections.

The mass transfer behavior is governed by a resistance $R_p$ with the same constitutive form as in the traditional 1D model of Pikal et al. (1983), with three empirical parameters ($R_p = a_0 + a_1 h_{d}/(1+a_2h_{d})$, Eq. (6)):

21 $$\begin{aligned} \dot{m}_{\mathrm{flow}} = \frac{A_{\mathrm{sub}}}{R_p} (p_{\mathrm{sub}} - p_{\mathrm{ch}}). \end{aligned}$$

where $p_{\mathrm{sub}}$ is the sublimation pressure of ice, computed in equilibrium with $T_{\mathrm{f}}$, and $p_{\mathrm{ch}}$ is the chamber pressure, and $A_{\mathrm{sub}}$ is the area available for sublimation (discussed further below). The frozen layer is taken to decrease in height proportionally to the change in mass, and is replaced by the dried layer:

22 $$\begin{aligned} h_{\mathrm{f}}= & \frac{m_{\mathrm{f}}}{m_{\mathrm{f,0}}} h_{\mathrm{f,0}} = \frac{m_{\mathrm{f}}}{m_{\mathrm{f,0}}} \frac{V_{\mathrm{fill}}}{A_p} \end{aligned}$$

23 $$\begin{aligned} h_{\mathrm{d}}= & h_{\mathrm{f,0}} - h_{\mathrm{f}} \end{aligned}$$

where $h_{\mathrm{f,0}}$ is the initial fill height.

**Table 5:** Summary of distinctions between the LC-IC, LC-DIC, and LC-DIF model variants

Acronym	Description	$A_{\mathrm{sub}}$	$Q_{\mathrm {vw-f}}$	$Q_{\mathrm {sh-f}}$	$Q_{\mathrm {sh-vw}}$
LC-IC	LC-IC: conduction only at ice–wall contact, curved front	$A_p + \alpha \sqrt{h_{\mathrm{d}}}$	$2\pi [Rh_{\mathrm{f}}K_{\mathrm {vw-f}}](T_{\mathrm{vw}}-T_{\mathrm{f}})$	$A_v K_{\mathrm {sh-f}}(T_{\mathrm{sh}}-T_{\mathrm{f}})$	$A_v\theta \sigma \left( T_{\mathrm{sh}}^4- T_{\mathrm{vw}}^4\right)$
LC-DIC	LC-DIC: conduction at wall through dry and ice, curved front	$A_p + \alpha \sqrt{h_{\mathrm{d}}}$	$2\pi [Rh_{\mathrm{f}}K_{\mathrm {vw-f}} + h_{\mathrm{d}}Sk_{\mathrm{d}}](T_{\mathrm{vw}}-T_{\mathrm{f}})$	$A_v K_{\mathrm {sh-f}}(T_{\mathrm{sh}}-T_{\mathrm{f}})$	$A_v\theta \sigma \left( T_{\mathrm{sh}}^4 -T_{\mathrm{vw}}^4\right)$
LC-DIF	LC-DIF: conduction through dry layer and ice at vial wall, flat front	$A_p$	$2\pi [Rh_{\mathrm{f}}K_{\mathrm {vw-f}} + h_{\mathrm{d}}Sk_{\mathrm{d}}](T_{\mathrm{vw}}-T_{\mathrm{f}})$	$A_p K_{\mathrm {sh-f}}(T_{\mathrm{sh}}-T_{\mathrm{f}})$	$(A_v-A_p) K_{\mathrm{sh-f}}(T_{\mathrm{sh}}-T_{\mathrm{f}})$

### LC-IC: conduction only at ice–wall contact, curved front

In Alexeenko et al. (2025), the vial-wall-to-frozen-layer heat transfer was given as

24 $$\begin{aligned} Q_{\mathrm {vw-f}} = 2\pi Rh_{\mathrm{f}} K_{\mathrm {vw-f}}(T_{\mathrm{vw}}-T_{\mathrm{f}}), \end{aligned}$$

which has the quirk that as drying concludes ($h_{\mathrm{f}} \rightarrow 0$), the thermal interaction between vial wall and product vanishes. This causes $T_{\mathrm{vw}}$ to reach much higher temperatures than are experimentally observed, even for reasonable values of $K_{\mathrm {vw-f}}$. This led to an assumption of Stefan-Boltzmann radiative heating from the top of the vial, with

25 $$\begin{aligned} Q_{\mathrm {sh-vw}} = A_v\theta \sigma (T_{\mathrm{sh}}^4-T_{\mathrm{vw}}^4) \end{aligned}$$

with $A_v$ vial outer cross section area, $\sigma$ the Stefan-Boltzmann constant, and $\theta =0.9$ an approximate emissivity, to stop runaway of $T_{\mathrm{vw}}$. This thermal interaction is already implicitly included in conventional empirical measurements of $K_{\mathrm {sh-f}}$, so together with the shelf-to-frozen-layer interaction

26 $$\begin{aligned} Q_{\mathrm {sh-f}} = A_v K_{\mathrm {sh-f}}(T_{\mathrm{sh}}-T_{\mathrm{f}}) \end{aligned}$$

some thermal radiation would be double-counted.

This original presentation also included a “curvature parameter” $\alpha$ in the sublimation area, with the notion that as drying proceeds the area available for sublimation would gradually increase. Hence rather than the vial inner cross section area $A_p$, $A_{\mathrm{sub}}$ in Eq. (21) was given as

27 $$\begin{aligned} A_{\mathrm{sub}} = A_{\mathrm{p}} + \alpha \sqrt{h_d} \end{aligned}$$

where the factor of $\alpha \sqrt{h_{\mathrm{d}}}$ would be about 0.1 cm$^{2}$ at the end of drying. In Fig. 7 this area is compared to a level set simulation, which indicates that only at the end of drying does $A_{\mathrm{sub}}$ deviate from $A_p$ (and in a complicated way), so a better approach would be to drop this term altogether.

**Fig. 10:**
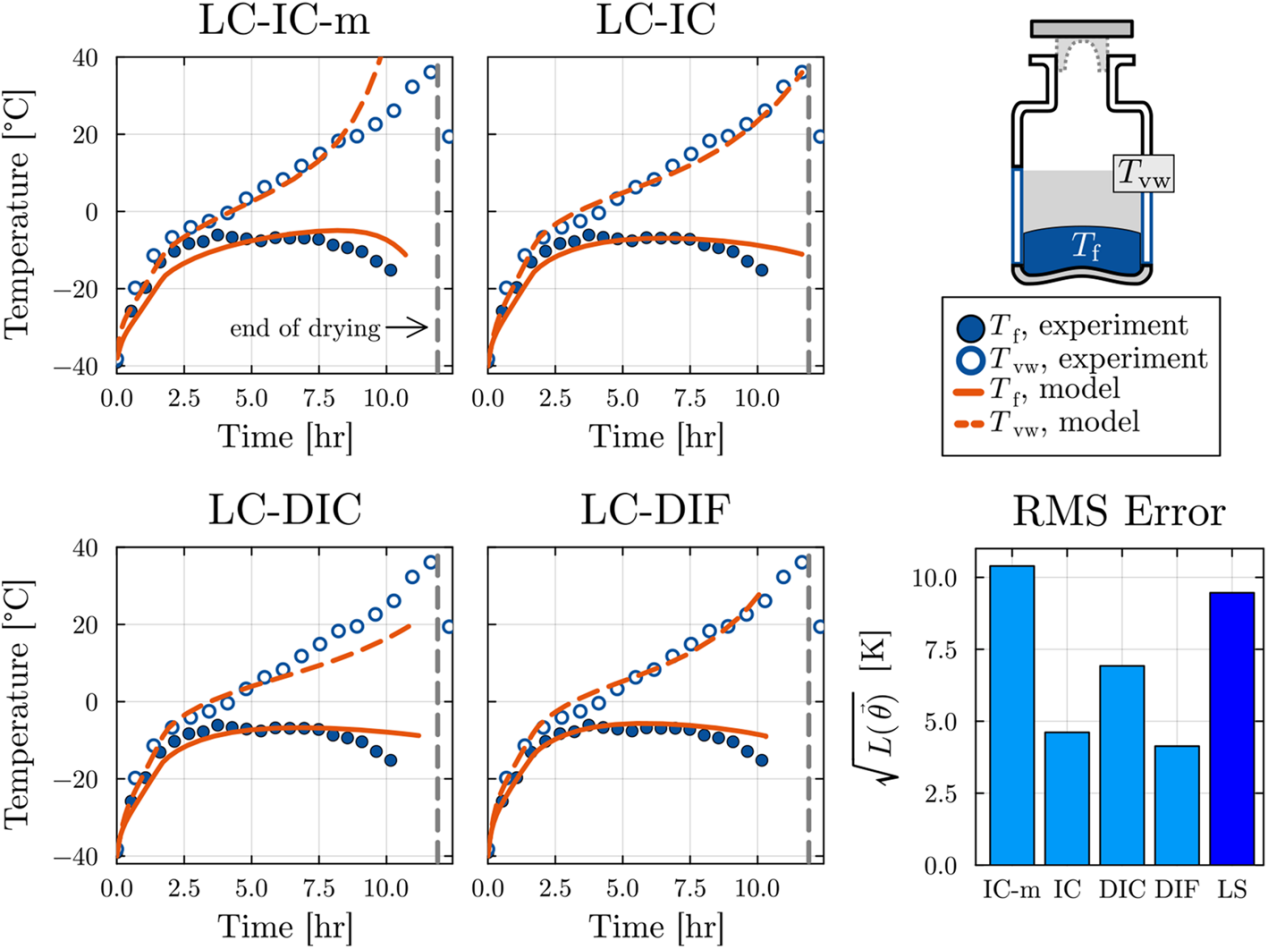
Original fit of LC-IC model in Alexeenko et al. (2025), denoted IC-m for its manual tuning, as well as least-squares fits of LC-IC, LC-DIC, and LC-DIF models to experimental temperatures for case M1 of Table 4. The square root of the “loss function” $L(\vec {\theta })$, which becomes essentially the root mean squared residual between experiment and model temperatures, is shown at right for the four fits as well as the LS model (see Fig. 5), as a measure of goodness of fit. For visual clarity, 40 experimental time points are shown, though measurements every minute (totaling over 600) are used in fitting

In Alexeenko et al. (2025), parameters were tuned manually, so in Fig. 10 the model results from there are denoted LC-IC-m to contrast with the least-squares fit we use here (described in Parameter fitting for lumped capacitance model).

### LC-DIC: conduction at wall through dry and ice, curved front

In early development of the level set model, we noticed that the level set model (which fully accounts for heat transfer through the dry layer) did not suffer from the same $T_{\mathrm{vw}}$ runaway as the LC-IC model. This suggested the inclusion of heat conduction through the dry layer in the lumped capacitance model. The vial-to-frozen-product heating term is thus given by:

28 $$\begin{aligned} Q_{\mathrm {vw-f}} = 2\pi \left[ R h_{\mathrm{f}} K_{\mathrm {vw-f}} + h_{\mathrm{d}} S k_{\mathrm{d}} \right] (T_{\mathrm{vw}} - T_{\mathrm{f}}), \quad \end{aligned}$$

This does require an estimate of the porous matrix’s thermal conductivity $k_{\mathrm{d}}$, which we estimate as the thermal conductivity of sucrose multiplied by the approximate volume fraction occupied by dry material (i.e., $k_{\mathrm{d}} = k_{\mathrm{sucrose}} \frac{c_{\mathrm{solid}}}{\rho _{\mathrm{solution}}}$). However, the magnitude of this heating term remains small, so the model is insensitive to uncertainty in $k_{\mathrm{d}}$.

The shape factor *S* in Eq. (28) is determined from an analytical solution for steady state heat conduction through a cylindrical dry layer (from the circular wall to the flat bottom with an adiabatic top), which can be tabulated offline and does not change over the course of simulation. *S* depends only on $\mathrm{Bi}_{\mathrm{d,rad}} = K_{\mathrm {vw-f}} R/k_{\mathrm{d}}$. To evaluate $S(\mathrm{Bi}_{\mathrm{d,rad}})$, solve first for a sequence of positive real eigenvalues $\lambda _n$ which satisfy

29 $$\begin{aligned} -\lambda _n J_1(\lambda _n) + \mathrm{Bi}_{\mathrm{d,rad}} J_0 (\lambda _n)=0, \end{aligned}$$

where $J_0$ and $J_1$ are the 0th and 1 st order Bessel functions of the first kind. *S* is then given by the following sum:

30 $$\begin{aligned} S = \sum \limits _{n=1}^\infty 2 \frac{J_1(\lambda _n)^2}{J_1(\lambda _n)^2 + J_0(\lambda _n)^2} \tanh {\lambda _n}, \end{aligned}$$

where the infinite sum can be comfortably approximated to within typical machine precision by around 200 terms. For values of $\mathrm{Bi}_{\mathrm{d,rad}}$ below $10^{-1}$, *S* is well approximated by $\mathrm{Bi}_{\mathrm{d,rad}}$, and for $\mathrm{Bi}_{\mathrm{d,rad}}$ larger than $10^4$, *S* approaches an asymptotic value of 4.032.

### LC-DIF: conduction through dry layer and ice at vial wall, flat front

To avoid double-counting radiation as in the LC-IC model, in the LC-DIF model, the shelf heating of both vial and frozen product are both governed by $K_{\mathrm {sh-f}}$ as measured by a gravimetric test, and the area across which this coefficient is applied is divided between the product area $A_p$ and the vial wall area $A_v-A_p$.

This leads to

31 $$\begin{aligned} Q_{\mathrm {sh-f}}= & A_pK_{\mathrm {sh-f}}(T_{\mathrm{sh}}-T_{\mathrm{f}}) \end{aligned}$$

32 $$\begin{aligned} Q_{\mathrm {sh-vw}}= & (A_v-A_p)K_{\mathrm {sh-f}}(T_{\mathrm{sh}}-T_{\mathrm{vw}}) \end{aligned}$$

Based on the LS model results shown above in Fig. 7, the effect of sublimation front curvature on mass transfer is small, particularly since a 5% effect is well within the uncertainty that is already present in many measurements of the mass transfer resistance $R_p$. We therefore drop the curvature parameter $\alpha$ altogether and revert to using the cross-sectional area inside the vial $A_p$ as the area available for sublimation.

### Empirical parameters

All of the existing models for microwave-assisted lyophilization, including those presented here, involve empirical parameters to be estimated from experiment. These are summarized in Table 6. Notably, prior works have treated the volumetric source term in the frozen layer (which we denote $Q'''_{\mathrm {RF-f}}$) directly as a fit parameter. Our approach here is to compute this from electromagnetic quantities as given in Eq. (10), such as the nominal input power an operator might control directly. This does not eliminate the fit parameter, but accommodates varying microwave power in a natural way.

**Table 6:** Summary of the empirical parameters to be estimated by fitting to experimental measurements for all the discussed models for microwave-assisted lyophilization. Each independent microwave-related fitting parameter is highlighted in red; i.e. Witkiewicz and Nastaj (2010) uses 5 microwave fit parameters and 1 mass transfer parameter, while LC-DIF uses 3 microwave fit parameters and 3 mass transfer parameters

Abbrev. & Source	Microwave heating parameters $Q^{\prime \prime \prime }_{\mathrm {RF-j}}=2\pi f\epsilon _0\epsilon _{j}^{\prime \prime }|E|_{\mathrm{j}}^{2}$	Mass transfer parameters
Witkiewicz and Nastaj (2010)	$K_{\mathrm{f}}(T_{\mathrm{f}})=\pi f\epsilon _0\epsilon ^{\prime \prime }_{\mathrm{f}} ={{\mu _{\mathrm{1,f}}}}T_{\mathrm{f}} +{{\mu _{\mathrm{2,f}}}}\, [=] \mathrm{Wm}^{-1} \mathrm{V}^{-2}$, MW absorption in frozen $K_{\mathrm{d}}(T_{\mathrm{d}}) = \pi f \epsilon _0 \epsilon ''_{\mathrm{d}} = {{\mu _{\mathrm{1,d}}}}T +{{\mu _{\mathrm{2,d}}}}\, [=] \mathrm{Wm}^{-1} \mathrm{V}^{-2}$, MW absorption in dry ${E}\,[=] \mathrm{Vm}^{-1}$, electric field intensity	$D_{\mathrm{eff}}\,[=] \mathrm{m}^{2} \mathrm{s}^{-1}$, effective diffusivity in dry layer ($R_p = \frac{h_{\mathrm{d}} RT}{D_{\mathrm{eff}}}$)
Park et al. (2021)	${{Q^{\prime\prime\prime}_{\mathrm {RF-f}}}} \,[=]\,\mathrm{W m}^{-3}$, volumetric microwave heating ($H_v$in their notation)	$T_{\mathrm{sub}}\,[=]\mathrm{K}$, temperature at sublimation front
Abdelraheem et al. (2022)	${{Q^{\prime \prime \prime}_{\mathrm {RF-f}}}}\,[=]\,\mathrm{Wm}^{-3}$, volumetric microwave heating ($\dot{Q}_{\mathrm{RF}}$in their notation)	$R_p(h_{\mathrm{d}})=a_0+\frac{a_1 h_{\mathrm{d}}}{1+a_2h_{\mathrm{d}}} \, [=] \mathrm{m s}^{-1}$, nonlinear mass transfer resistance
LS, this work	${{K_{\mathrm {vw-f}}}\,[=]\mathrm{WK}^{-1} \mathrm{m}^{-2}}$, heat transfer between vial wall and frozen layer ${B_{\mathrm{f}}}=\frac{|E|_{\mathrm{f}}^{2}}{P_{\mathrm{MW}}/N_{\mathrm{vial}}}\,[=]\Omega \mathrm{m}^{-2}$, electric field in frozen layer due to nominal power per vial ${B_{\mathrm{vw}}}\,[=]\Omega \mathrm{m}^{-2}$, electric field in vial wall due to nominal power per vial	*l*(*r*, *z*) porous structure constant
LC-IC, Alexeenko et al. (2025)	${K_{\mathrm {vw-f}}}$	$R_p(h_{\mathrm{d}})$ ${{\alpha }}\,[=]\mathrm{m}^{3/2}$, apparent sublimation front curvature
	$${B_{\mathrm{f}}}$$	
	$${B_{\mathrm{vw}}}$$	
LC-DIF, this work	${K_{\mathrm {vw-f}}}$	$R_p(h_{\mathrm{d}})$
	$${B_{\mathrm{f}}}$$	
	$${B_{\mathrm{vw}}}$$	

### Parameter fitting for lumped capacitance model

In comparing to experiment, there are a total of 4 parameters to be determined empirically in the conventional model for primary drying:

- $K_{\mathrm {sh-f}}$: heat transfer coefficient from shelf to frozen layer; often measured separately with a gravimetric test
- $a_0, a_1, a_2$: mass transfer resistance coefficients, in the form $R_p = a_0 + \frac{a_1 h_d}{1 + a_2 h_d}$.

In the lumped capacitance model, an additional 3 empirical parameters are added (or 4 in the case of LC-IC and LC-DIC):

- $B_{\mathrm{f}}$ and $B_{\mathrm{vw}}$: the relative electric field strength in the frozen layer and vial wall, respectively; equivalent to one parameter for overall absorption, and one for partitioning between ice and glass
- $K_{\mathrm {vw-f}}$: heat transfer coefficient from vial wall to frozen layer
- $\alpha$: a mass transfer-related parameter

Various experimental methods exist for measuring the conventional fitting parameters, depending on the availability of various PAT tools (Pikal et al. 2005; Shivkumar et al. 2019; Tchessalov et al. 2022; Srinivasan et al. 2023). For this work, a least-squares fit is employed to match temperature predictions by the model against experimentally measured temperatures. This least-squares criterion is augmented by adding a term comparing the model’s predicted end of drying to the end of drying determined from the Pirani sensor pressure measurement. The parameters used in the microwave heating model are tuned by a similar procedure, with the addition of comparison against vial wall temparatures when experimentally available. The full objective function used as a criterion for parameter tuning is then as follows:

33 $$\begin{aligned} L(\vec {\theta }) = \frac{1}{n_1} \sum \limits _{{i=1}}^{n_1} \left( T_{{f,i}} - \hat{T}_{{f,i}}\right) ^2 + \frac{C_2}{n_2} \sum \limits _{{i=1}}^{n_2} \left( T_{\mathrm{vw,i}} - \hat{T}_{\mathrm{vw,i}}\right) ^2 + C_3 \left( t_{\mathrm{end}} - \hat{t}_{\mathrm{end}} \right) ^2 \end{aligned}$$

where $C_2$ is set to 1 if vial wall temperatures are available and 0 otherwise, and $C_3$ is arbitrarily given a value of $0.1 \frac{K^2}{hr^2}$ to balance the time objective against temperature objective. Values with a hat (e.g. $\hat{T}_{\mathrm{vw}}$) are used to denote model predictions computed with the set of fit parameter values $\vec {\theta }$ at times *i* corresponding to experimental measurements. We use a differing $n_1$ and $n_2$ (count of temperature measurements for frozen layer and vial wall, respectively) because the lumped capacitance model cannot describe product temperature behavior near end of drying, as is the case for the traditional model applied to conventional lyophilization; product temperatures after a sudden rise are manually excluded from the fit, while vial wall temperatures can still be used.

Exploratory parameter fitting indicates that estimates of $K_{\mathrm {vw-f}}$ are strongly dependent on estimates of the vial wall temperature, which was not explicitly measured for case M2, SM1, or any of the microwave vacuum drying cases discussed in literature. In cases when vial wall temperature was measured, it can be heuristically observed that, towards the end of drying, temperatures in the frozen layer diverge from a nearly-flat trajectory and approach vial wall temperatures. This observation is reinforced by the 2D simulation results, as discussed above in Level set model results. On these grounds, for cases without vial wall temperature measurement, the measured temperature in the frozen layer at the end of primary drying, just before turning off microwave power, is treated as a measurement of the vial wall temperature and included in the objective function Eq. (33), alongside the frozen layer temperatures before the temperature rise.

We minimize the loss function not by explicitly constructing the loss function Eq. (33), but by solving the corresponding NonlinearLeastSquaresProblem with NonlinearSolve.jl. To allow the fitting procedure to run over all real numbers, the package TransformVariables.jl is used to map the space of real numbers to appropriately-dimensional quantities, constraining the fit to physically reasonable (e.g. nonnegative) values. In some cases the “reasonable” space was limited to a span of 6 orders of magnitude about a guess value of a given parameter, e.g. $1 \times 10^{-3}$ to $1 \times 10^{3}$ times an expected estimate, rather than the span of all positive numbers.

## Results and discussion - updated simplified model

### Comparison of lumped capacitance model variants

**Fig. 11:**
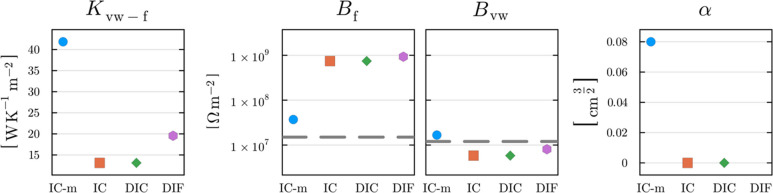
Best-fit values of fitting parameters for the variants of the LC model, compared to the original values given in Alexeenko et al. (2025) (denoted IC-m). Dotted lines for $B_{\mathrm{f}}$ and $B_{\mathrm{vw}}$ indicate order-of-magnitude estimates from the electromagnetic simulation in Electric field strength and parameters; differences of a few orders of magnitude are unsurprising since the electromagnetic behavior is highly uncertain (both in terms of physical properties and simulation simplifications). Since $\alpha$ is dropped entirely from the LC-DIF model, no point is shown for its value

To assess the relative validity of the three model variants described in LC-IC: conduction only at ice–wall contact, curved front, LC-DIC: conduction at wall through dry and ice, curved front, and LC-DIF: conduction through dry layer and ice at vial wall, flat front, we show the original fit of the LC-IC model presented in Alexeenko et al. (2009) and show a least-squares fit of each model variant to the same experimental case. Temperature fits (and the value of the loss function *L* given in Eq. (33) are shown in Fig. 10, with resulting fit parameters in Fig. 11; note that since $\alpha$ is left out of LC-DIF, it is shown without a value. The LS model realization of Fig. 5 shares fit parameters with the LC-DIF model, and its comparison to experiment is also shown in Fig. 10.

Although the best-fit parameters are somewhat different when fitting with different variants of the model, the models achieve a similar level of fit. Given the lack of significant curvature shown with the LS model in Curvature assessment, as well as the best-fit values close to 0 in Fig. 11, it is reasonable to drop the parameter $\alpha$. Thus the LC-DIF is able to achieve comparable goodness of fit without one of the four fitting parameters, and we recommend it be preferred to LC-IC model presented in Alexeenko et al. (2025).

### LC-DIF model behavior: energy budget analysis

**Fig. 12:**
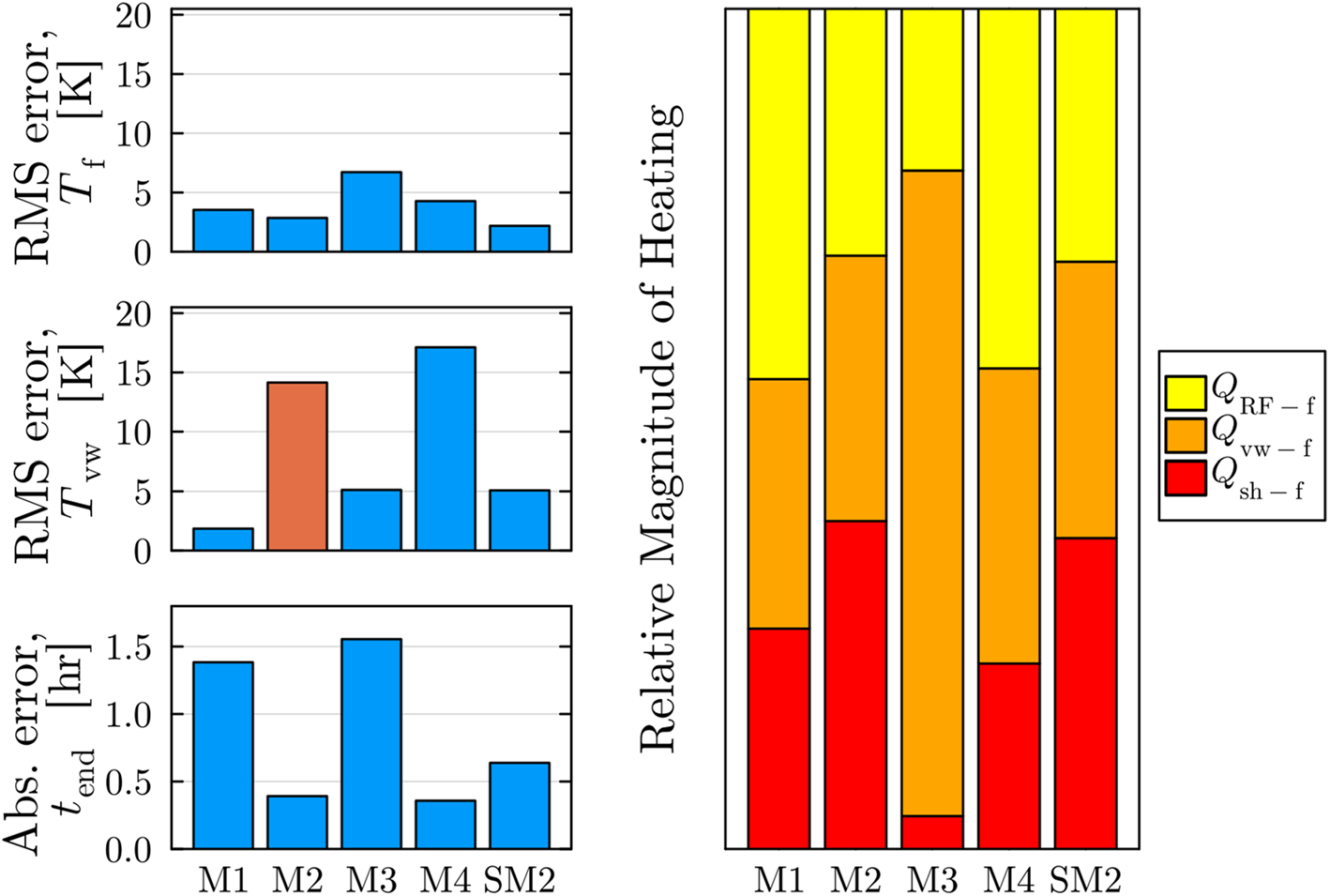
Left: the error between experimental measurements and LC-DIF model predictions; the orange bar for M2 is to distinguish that for this experimental case, $T_{\mathrm{vw}}$ was not measured, so a final-time value of $T_{\mathrm{f}}$ is used as a substitute. Right: the relative magnitude of the three heating modes which directly contribute to sublimation, across experimental cases

Table 7 shows best fit parameters across the experimental cases listed in Table 4, noting that for M2 no parameters are fit and for M4 $K_{\mathrm {vw-f}}$ is taken equal to its value for M1. The left of Fig 12 shows the experimental agreement, broken out by error in $T_{\mathrm{f}}$, $T_{\mathrm{vw}}$, and $t_{\mathrm{end}}$ respectively, noting that for M2 vial wall temperature measurements are not available and so the fit uses a highest measured value of $T_{\mathrm{f}}$ as an approximate final value of $T_{\mathrm{vw}}$. Cases M1 and M2 differ only by fill volume (from 5mL to 3 mL), so the good fit in M2 with parameters matched to M1 confirms that the model is well-behaved and that the three empirical parameters port well at least across large and medium fill volumes. Cases M3 and M4 are at much higher microwave frequency (18 GHz vs. 8 GHz), which makes it likely that electromagnetic behavior changes, so change in $B_{\mathrm{f}}$ and $B_{\mathrm{vw}}$ is unsurprising. The much larger best-fit $K_{\mathrm {vw-f}}$ for case M3 does not have an obvious explanation. Before primary drying in case M3 (a closed-loop control experiment), the product in the chamber was thermally cycled to tune the PID controller gains, and one possible hypothesis is that this thermal cycling caused annealing which changed the mass transfer resistance or decreased the thermal resistance between the porous cake and the glass wall.

Three main heat transfer mechanisms contribute directly to the sublimation process in the ice:

- $Q_{\mathrm {sh-f}}$, shelf heating of the frozen layer
- $Q_{\mathrm {vw-f}}$, heat transfer from the vial wall into the frozen layer (due to elevated temperatures in the vial wall, caused by volumetric heating in the glass)
- $Q_{\mathrm {RF-f}}=Q'''_{\mathrm {RF-f}}V_{\mathrm{f}}$, direct volumetric heating of the frozen layer by the microwave field

In the right of Fig. 12, these three contributions are computed in the model and integrated over time, then normalized by their total to compare across experimental cases. Clearly none of these three heat transfer mechanisms can, in general, be neglected—all contribute. The smallest contribution visible here is the apparent 5% contribution of $Q_{\mathrm {sh-f}}$ in case M3; it is small simply because the closed loop setpoint temperature (used to adjust microwave heating for that experiment) is close to the shelf temperature setpoint, and in fact has a net negative contribution because the shelf set point is below the product set point.

**Table 7:** Comparison of LC-DIF best fit parameters across 5 microwave-assisted lyophilization experiments. Case M2 is deliberately given identical parameters to M1. In the electromagnetic simulation (Electric field strength and parameters), $B_{\mathrm{f}}$ and $B_{\mathrm{vw}}$ are estimated as $1.50 \times 10^{7} \Omega \mathrm{m}^{-2}$ and $1.20 \times 10^{7} \Omega \mathrm{m}^{-2}$ respectively, although order of magnitude differences are unsurprising

	$K_{\mathrm {vw-f}}, \mathrm{WK}^{-1} \mathrm{m}^{-2}$	$B_{\mathrm{f}}, \Omega \mathrm{m}^{-2}$	$B_{\mathrm{vw}}, \Omega \mathrm{m}^{-2}$
M1	18.5	$8.74 \times 10^{8}$	$8.12 \times 10^{6}$
M2	18.5	$8.74 \times 10^{8}$	$8.12 \times 10^{6}$
M3	209	$5.88 \times 10^{7}$	$4.35 \times 10^{6}$
M4	18.5	$2.04 \times 10^{8}$	$3.84 \times 10^{6}$
SM2	6.17	$8.40 \times 10^{8}$	$9.66 \times 10^{6}$

**Fig. 13:**
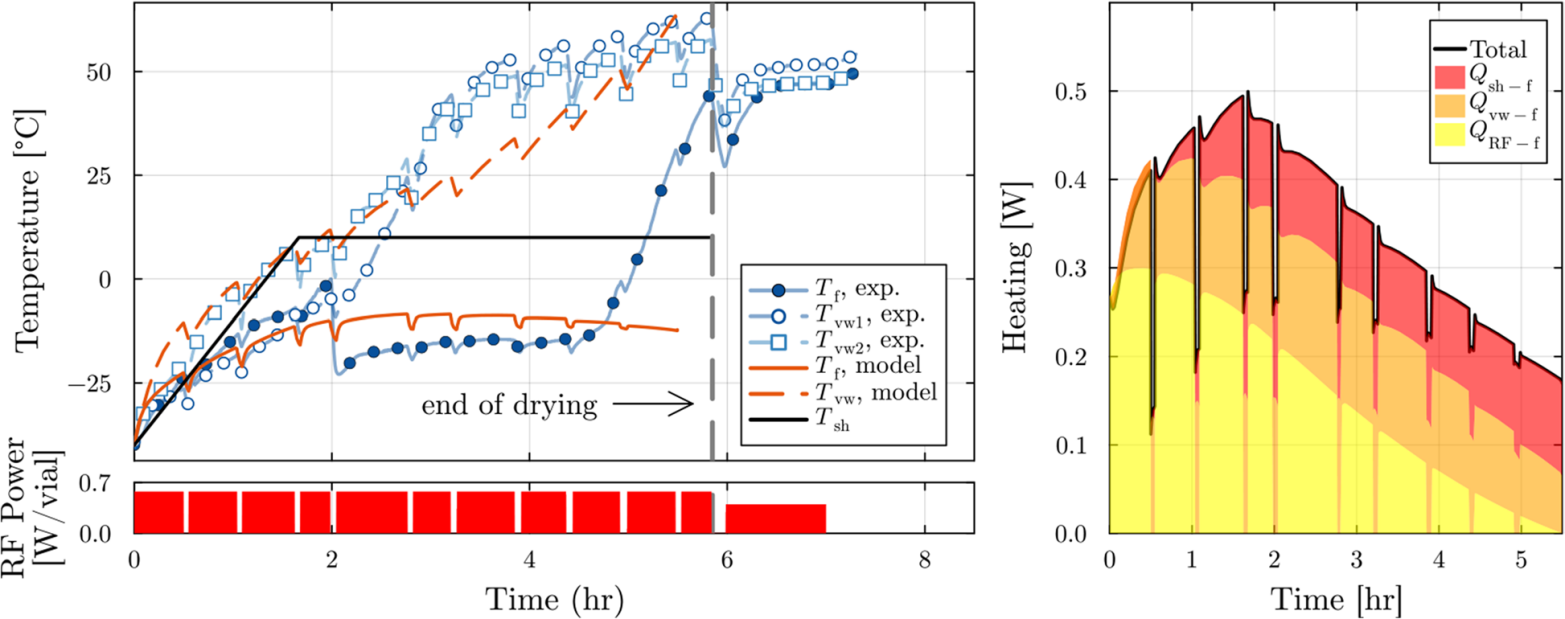
Left: temperatures measured in experiment and predicted by model for the experimental case denoted M4, accompanied just below by the time-dependent microwave output (with on-off behavior dictated by manual adjustment). Right: the magnitudes over time of the heating modes predicted by the LC-DIF model

**Fig. 14:**
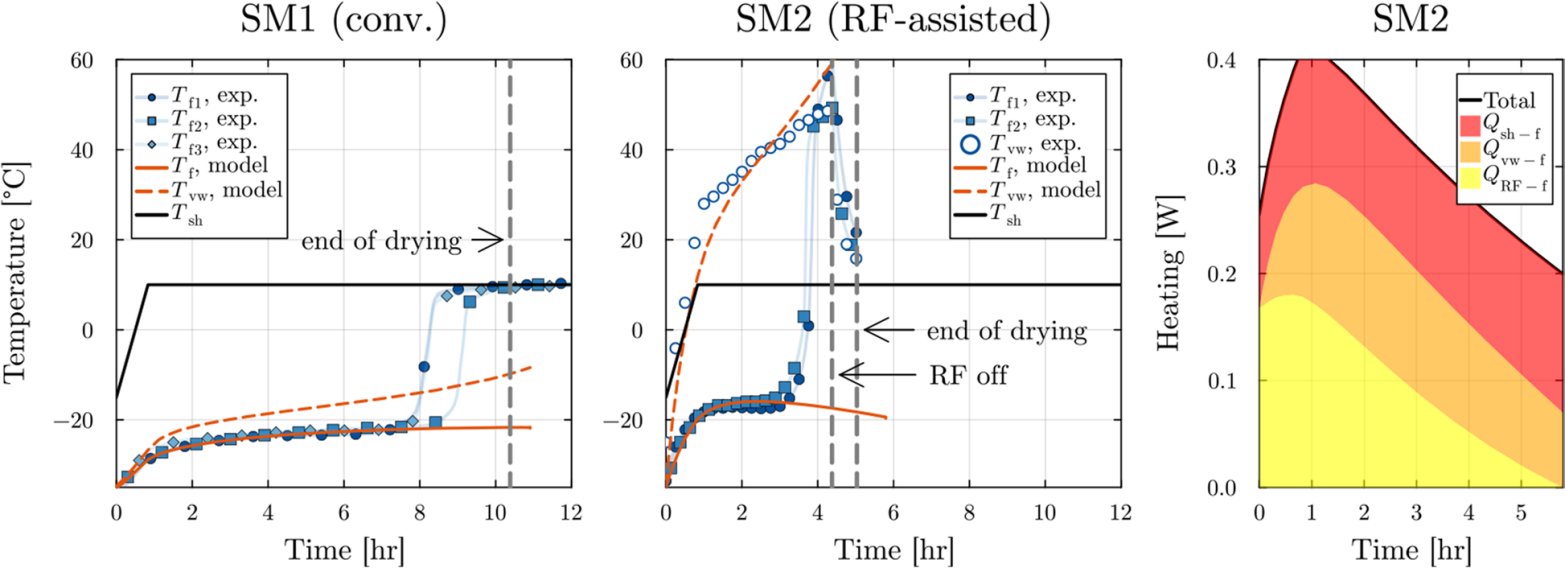
Left: experimental and model temperatures for case SM1, conventional lyophilization of a sucrose-mannitol formulation. Center: experimental and model temperatures for case SM2, RF-assisted lyophilization of the sucrose-mannitol formulation. Right: magnitude of heating modes over time predicted by the LC-DIF model for case SM2

In Figs. 13 and 14 we show experimental and model temperatures for experimental cases M4, SM1, and SM2. Corresponding figures for cases M1, M2, and M3 are in the appendix, Figs. 17, 18, and 19. In each of these figures, the left facet compares model predicted temperatures to experimental measurements and the right facet shows the model predicted heat transfer modes over time. In all of these cases, the microwave-related heat transfer decreases near the end of drying: direct microwave heating of the product $Q_{\mathrm {RF-f}}$ goes to zero as the volume of ice goes to zero, and heating from the vial wall $Q_{\mathrm {vw-f}}$ decreases as the direct contact between ice and vial wall goes to zero.

In case M4 (Fig 13), the vial with product temperature probe underwent meltback: at about $t=2\mathrm {\,hr}$, $T_{\mathrm{f}}$ exceeded the eutectic melt temperature, then dropped suddenly by nearly 20 K, and afterward the sample had a visibly different shape from all the other samples dried in that cycle, including those for which vial wall temperature was measured. By changing the cake shape, meltback causes radically different mass transfer behavior than the model here can accommodate, so measured product temperatures are not considered in the fit after the meltback event. Although this leaves a persistent offset between measured and modeled temperatures, the model does approximate the magnitude of the sawtooth temperature swings caused by turning microwave power off and on at intervals.

For case SM1, which is a conventional lyophilization cycle, the left of Fig. 14 shows that the lumped capacitance model (with microwave heating set to zero) describes experimental temperatures and drying time about as well as the traditional 1D pseudosteady model, which is unsurprising given its similar structure. It additionally predicts that, until primary drying is completely finished, the vial wall temperature does not get too far from the product temperature. The center of Fig. 14 shows that although the LC-DIF model with least-squares fit has similar overprediction of drying time to the LC-IC model (see Fig. 5 of Alexeenko et al. (2025)), it can match temperature predictions before the rise at $t=3\,\mathrm{hr}$. This overprediction in drying time could suggest that the mass transfer resistance is changing due to the higher product temperatures, a phenomenon often described as “microcollapse” (Bhambhani et al. 2021; Srinivasan et al. 2023). The very high observed temperatures of the vial wall lead to a smaller best-fit value of $K_{\mathrm {vw-f}}$, shown in Table 7.

### LC-DIF model applied to microwave vacuum dryers

Bhambhani et al. (2021) provide some experimentally measured temperatures in their Fig. 5 for a microwave vacuum dryer setup, and likewise Gitter et al. (2019) provide temperatures in their Fig. 1 for another similar dryer. These experiments are listed in Table 4 as S1-S2 and F1-F4, respectively. In all of these experiments, the microwave vacuum dryer is not equipped with temperature-controlled shelves like a traditional lyophilizer. To apply the LC-DIF model to their experiments therefore takes a little extra consideration. Even in a traditional lyophilizer, some amount of the heat transfer which is accounted for as coming from the shelf actually comes from radiative heat exchange with other parts of the lyophilizer (Srisuma et al. 2024; Tchessalov et al. 2022), which is responsible for the so-called “edge effect”. In a microwave vacuum dryer without controlled shelf temperatures, this same radiative effect will cause some small amount of radiative heat transfer, so in fitting the LC-DIF model to these cases we must also fit $K_{\mathrm {sh-f}}$.

**Fig. 15:**
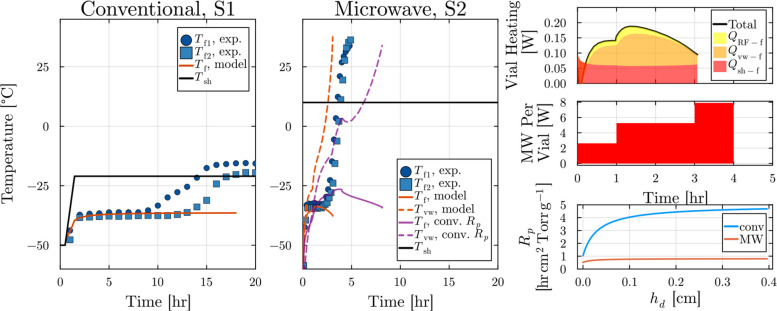
Fit of LC-DIF model to cases S1 and S2, corresponding to Fig. 5a and b respectively of Bhambhani et al. (2021). Two representative temperature series are chosen from their six reported series. Purple temperature trace at center reuses $R_p$ as fit to conventional, while orange fits a new $R_p$. At right, the model’s predicted heat transfer modes and applied microwave heating over time are shown, as well as the best-fit $R_p$ for both the conventional and microwave cycles

In principle, we could use S1, which is a conventional lyophilization cycle, to fit for $R_p$ and reuse the values for case S2. However, in agreement with Bhambhani et al. (2021)’s assessment, matching $R_p$ between the two cases leads to a factor of 2 overestimation of cycle time, as shown by the purple temperature trace of Fig. 15. The orange temperature trace, which fits much more closely, uses a simultaneous fit for $K_{\mathrm {sh-f}}$, $R_p$, and microwave parameters—the best-fit $R_p$ is much smaller than for the conventional cycle, which could be a temperature-related change in the porous structure.

For cases F1, F2, and F4 from Gitter et al. (2019), we likewise do a simultaneous fit of both conventional and microwave-related parameters. Their only chamber pressure measurement is a Pirani gauge, which is sensitive to the gas composition in the chamber, so the actual pressure is uncertain. All of the resulting fit parameters are in Appendix Table 8. LC-DIF fits to cases F1 and F2 are shown in the appendix, Figs. 20 and 21.

**Fig. 16:**
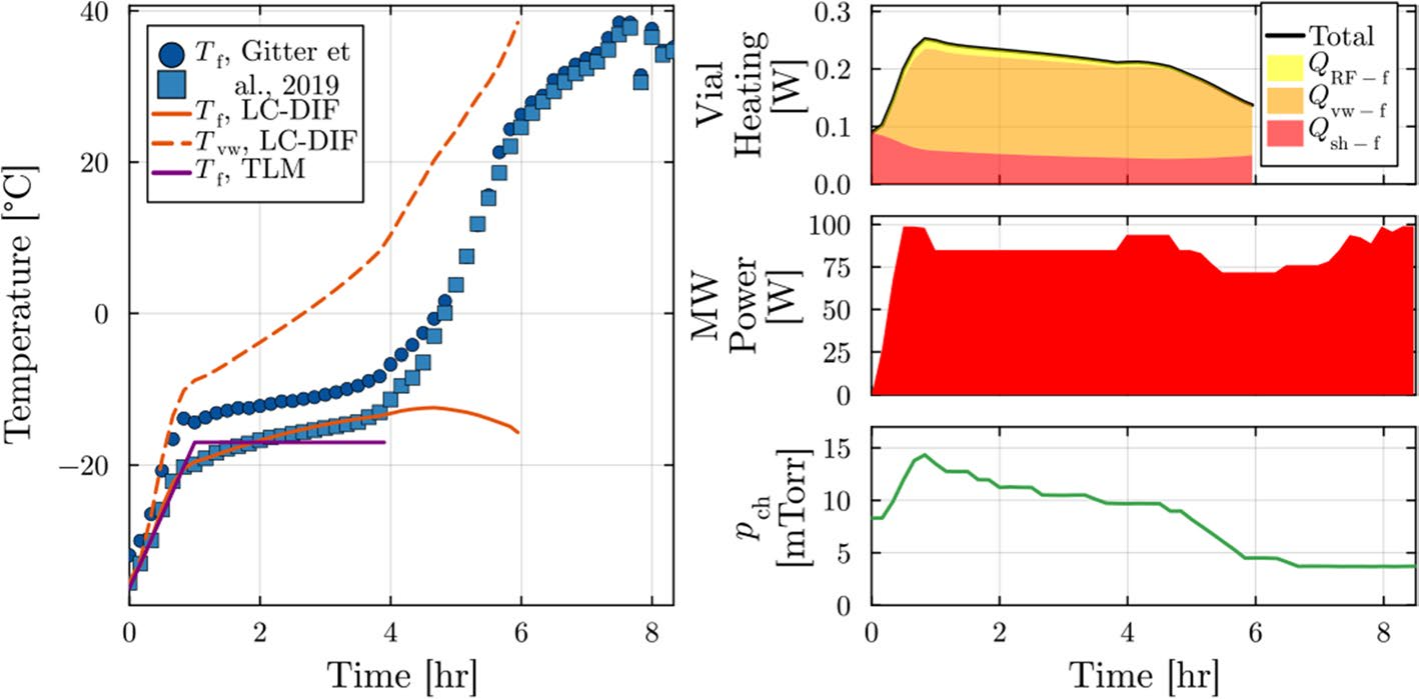
Fit of LC-DIF model to Fig. 1c of Gitter et al. (2019), plus the model results given in Fig. 4 of Park et al. (2021). The right center and right lower plots give the microwave heating and chamber pressure as reported by Gitter et al. (2019)

**Fig. 17:**
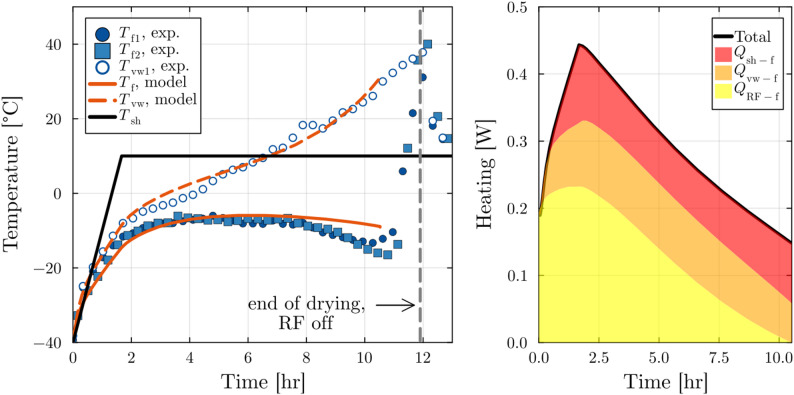
At left, temperatures measured in experiment and predicted by model for the experimental case denoted M1. At right, the magnitudes over time of the heating modes predicted by the model

**Fig. 18:**
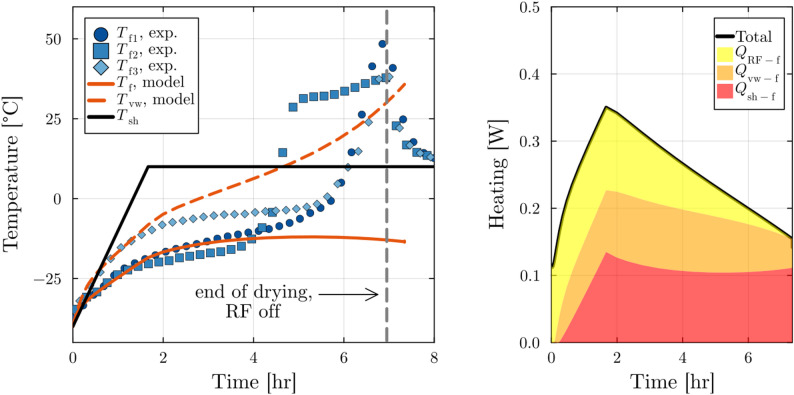
At left, temperatures measured in experiment and predicted by model for the experimental case denoted M2. At right, the magnitudes over time of the heating modes predicted by the model

**Fig. 19:**
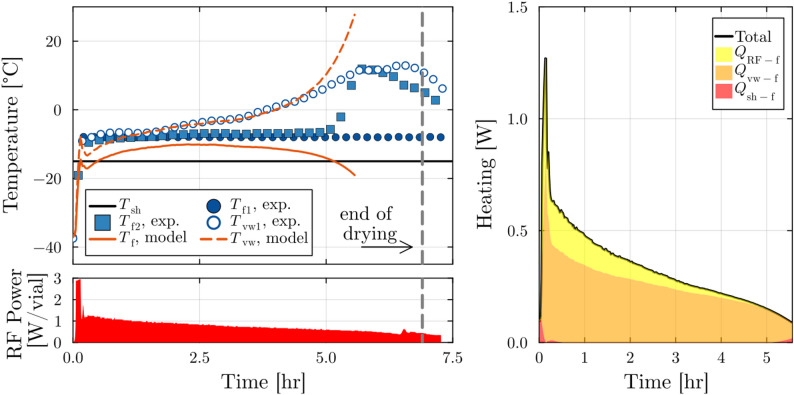
At left, temperatures measured in experiment and predicted by model for the experimental case denoted M3, accompanied by the RF power output dictated by the closed loop controller. At right, the magnitudes over time of the heating modes predicted by the model

**Fig. 20:**
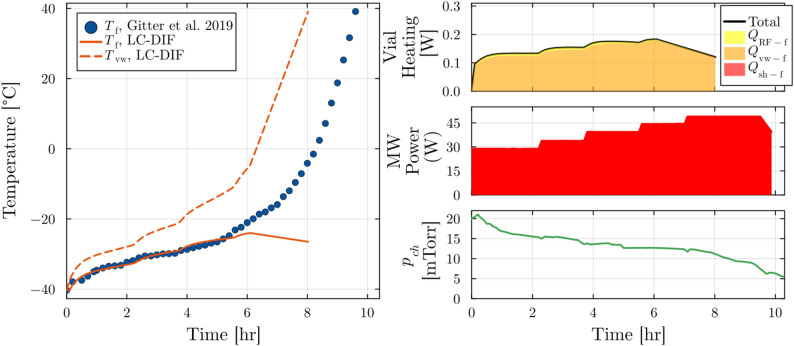
LC-DIF model fit to temperature data reported in Gitter et al. (2019), Fig. 1a

**Fig. 21:**
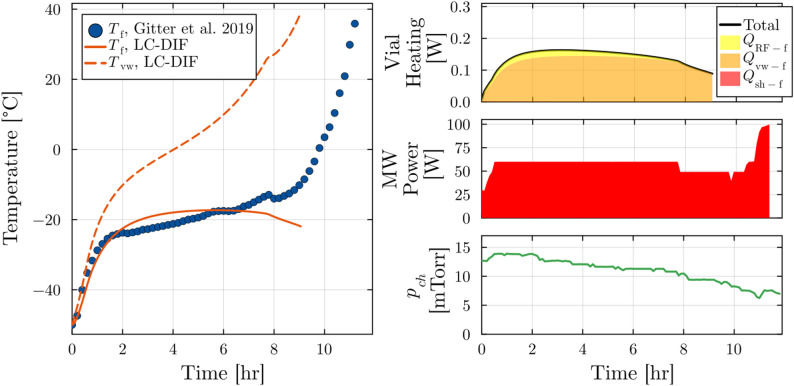
LC-DIF model fit to temperature data reported in Gitter et al. (2019), Fig. 1b

Figure 16 shows the LC-DIF fit to case F4, alongside the reported model heat transfer modes, reported microwave heating, and reported pressure. For reference, the model results shown in Park et al. (2021)’s Fig. 4 are shown on the same axes, denoted TLM (for “thermally limited model”). Among other fit parameters, they set $T_{\mathrm{sub}}=-17 ^{\circ }\mathrm{C}$ (which corresponds to a sublimation pressure of about 1000mTorr, compared to chamber pressure of about 10mTorr) and use a fill height (denoted $h_{\mathrm{f0}}$ here and *L* there) of 4.2 cm instead of the 2.3 cm that can be deduced from reported fill volume and vial size.

Park et al. (2021) builds on the models presented in Velardi and Barresi (2008) to provide two models for microwave-assisted freeze drying, one detailed and one simplified. The simplified model admits an analytical solution, which is presented in Srisuma et al. (2023). The bulk of their analytical work there is to provide a solution for the early-time behavior of lyophilization, when heat transfer is transient (e.g. in the first hour or so, before a pseudosteady state is reached). Equation 21 of Park et al. (2021) gives the mass balance as governed exclusively by heat transfer behavior. This is equivalent to letting $R_p = 0$, or assuming that the mass transfer is limited solely by the heat transfer resistance. Mathematically, this has the consequence that the sublimation temperature is taken as a constant, and so the analytical solution to the bulk of the drying phase is exceedingly simple—so simple that no equation is given there for the temperature, only for the position of the sublimation front. As a consequence, the addition of microwave heating to this model does not impact the temperature of the frozen layer—mathematically, it increases the drying rate, but cannot increase the temperature since temperature is fixed. This directly contrasts the experimental results seen in Alexeenko et al. (2025), where the addition of microwave heating increases the temperatures observed during lyophilization.

## Conclusion

We developed a 2D level set (LS) model for simulating microwave-assisted freeze drying in a vial. Based on that model’s results, we recommend revisions to the (0D) lumped capacitance (LC) model of Alexeenko et al. (2025), and with electromagnetic simulations we provide order-of-magnitude estimates of empirical parameters introduced there. The LS and LC-DIF model implementations in Julia are provided as open-source code under the MIT license on GitHub and at Wheeler (2026a); Wheeler et al. (2026)

1. The empirical parameters $B_{\mathrm{f}}$ and $B_{\mathrm{vw}}$, which relate nominal input power to electric field in the frozen layer and vial wall respectively, can be estimated as $1 \times 10^{7} \Omega \mathrm{m}^{-2}$, but may vary by a few orders of magnitude. These parameters *can* depend on the lyophilizer geometry, microwave setup, electric field frequency, vial geometry, fill volume, and formulation, but in practice may not be sensitive to all these factors in all cases.
2. The LC models make clear that in microwave-assisted lyophilization, as physically realized in Alexeenko et al. (2025), heat conduction from the vial wall to the frozen product cannot be neglected, because the vial wall itself undergoes microwave heating. This is in contrast to conventional freeze drying, where radial conduction can be neglected (or is implicitly included when empirically measuring $K_{\mathrm {sh-f}}$). Shelf heating and direct microwave heating of the product are also significant and should be considered.
3. High temperatures observed in the product near or at the end of primary drying tend to correspond to vial wall temperatures, which assists in constraining the fit for $K_{\mathrm {vw-f}}$, $B_{\mathrm{f}}$, and $B_{\mathrm{vw}}$.
4. The addition of microwave heating does not, on its own, cause enough sublimation front curvature to impact the mass transfer behavior of lyophilization in a vial.
5. For activities such as parameter estimation and cycle development of microwave lyophilization in vials which require a simple, cheap model, the LC-DIF model presented here should be used. The LC-IC model originally given in Alexeenko et al. (2025) has an extra parameter to fit and is less accurate, while the analytical model of Srisuma et al. (2023) neglects mass transfer resistance.
6. Spatially varying pore structures may, even in conventional lyophilization, cause significant sublimation front curvature. This curvature may manifest as a distinct value of $R_p$ from a planar front, but the traditional nonlinear model may still fit it well. The LS model is well-suited to further investigation of this behavior, since it is cheaper than full CFD models and can describe a nonplanar front (unlike 0D or 1D models).
7. Imperfect placement of thermal probes, which is often inevitable in experiment, can cause the temperature rise often observed before the end of primary drying.

## Data Availability

The lyophilization process data and analysis scripts used (including to generate all figures) during the current study are available at Wheeler (2026b); simulation code is contained in Wheeler et al. (2026); Wheeler (2026a).

## Appendix A Other examples of LC-DIF model compared to experiment

**Table 8:** Both conventional and microwave-related fit parameters for fits to the temperature data reported in Fig. 5a-b of Bhambhani et al. (2021) and Fig. 1 of Gitter et al. (2019). Figure 5b of Bhambhani et al. (2021) is a conventional cycle, so the microwave fit parameters $K_{\mathrm {vw-f}}$, $B_{\mathrm{f}}$, and $B_{\mathrm{vw}}$ are not used

	$K_{\mathrm {sh-f}}$	$a_{\mathrm{0}}$	$a_{\mathrm{1}}$	$a_{\mathrm{2}}$	$K_{\mathrm {vw-f}}$	$B_{\mathrm{f}}$	$B_{\mathrm{vw}}$
	$\mathrm{WK}^{-1}\ \mathrm{m}^{-2}$	$\mathrm {h\ cm}^{2} \mathrm {Torr\ g}^{-1}$	$\mathrm {h\ cmTorr\ g}^{-1}$	$\mathrm{cm}^{-1}$	$\mathrm{WK}^{-1}\ \mathrm{m}^{-2}$	$\Omega \mathrm{m}^{-2}$	$\Omega \mathrm{m}^{-2}$
Bhambhani 2021 Fig. 5b	8.56	1.05	125	31.7			
Bhambhani 2021 Fig. 5a	7.39	0.515	16.0	54.6	49.4	$7.90 \times 10^{8}$	$2.99 \times 10^{6}$
Gitter 2019 Fig. 1a	0.0246	2.28	9.54	0.002 76	70.5	$9.37 \times 10^{6}$	$3.19 \times 10^{5}$
Gitter 2019 Fig. 1b	0.0324	2.61	104	5.85	30.9	$8.74 \times 10^{6}$	$1.84 \times 10^{5}$
Gitter 2019 Fig. 1c	5.17	5.18	25.0	0.0118	38.8	$8.76 \times 10^{6}$	$1.39 \times 10^{5}$
